# Host plant adaptation in the polyphagous whitefly, *Trialeurodes vaporariorum*, is associated with transcriptional plasticity and altered sensitivity to insecticides

**DOI:** 10.1186/s12864-019-6397-3

**Published:** 2019-12-19

**Authors:** Adam Pym, Kumar Saurabh Singh, Åsa Nordgren, T. G. Emyr Davies, Christoph T. Zimmer, Jan Elias, Russell Slater, Chris Bass

**Affiliations:** 1 College of Life and Environmental Sciences, Biosciences, University of Exeter, Penryn Campus, Penryn, Cornwall UK; 2 Department of Biointeractions and Crop Protection, Rothamsted Research, Harpenden, UK; 3 Syngenta Crop Protection, Werk Stein, Schaffhauserstrasse, Stein, Switzerland

**Keywords:** Polyphagy, Resistance, Whitefly, Xenobiotic, Insecticide

## Abstract

**Background:**

The glasshouse whitefly, *Trialeurodes vaporariorum*, is a damaging crop pest and an invasive generalist capable of feeding on a broad range of host plants. As such this species has evolved mechanisms to circumvent the wide spectrum of anti-herbivore allelochemicals produced by its host range. *T. vaporariorum* has also demonstrated a remarkable ability to evolve resistance to many of the synthetic insecticides used for control.

**Results:**

To gain insight into the molecular mechanisms that underpin the polyphagy of *T. vaporariorum* and its resistance to natural and synthetic xenobiotics, we sequenced and assembled a reference genome for this species. Curation of genes putatively involved in the detoxification of natural and synthetic xenobiotics revealed a marked reduction in specific gene families between this species and another generalist whitefly, *Bemisia tabaci*. Transcriptome profiling of *T. vaporariorum* upon transfer to a range of different host plants revealed profound differences in the transcriptional response to more or less challenging hosts. Large scale changes in gene expression (> 20% of genes) were observed during adaptation to challenging hosts with a range of genes involved in gene regulation, signalling, and detoxification differentially expressed. Remarkably, these changes in gene expression were associated with significant shifts in the tolerance of host-adapted *T. vaporariorum* lines to natural and synthetic insecticides.

**Conclusions:**

Our findings provide further insights into the ability of polyphagous insects to extensively reprogram gene expression during host adaptation and illustrate the potential implications of this on their sensitivity to synthetic insecticides.

## Background

The greenhouse whitefly, *Trialeurodes vaporariorum* is an economically important agricultural pest found in temperate environments across the globe [1]. The nymph and adult stages of this species cause damage via direct feeding on the plant phloem, the transmission of plant viruses and the production of honeydew that supports the growth of sooty mould [2, 3]. *T. vaporariorum* feeds on more than 275 different plant species encompassing a range of vegetable, fruit and ornamental crops. Utilising a broad host range represents a significant challenge to polyphagous insects like *T. vaporariorum* as they encounter a wide spectrum of secondary metabolites produced by their hosts as an anti-herbivore defence mechanism. These allelochemicals can be extremely diverse [4] encompassing alkaloids, amines, cyanogenic glucosides, glucosinolates, non-protein amino acids, organic acids, terpenoids, phenolics, quinones, polyacetylenes, and peptides [5, 6]. Recent work has provided evidence that generalist insects can respond to secondary metabolites produced by host plants by inducing changes in gene expression that provide greater fitness on a specific host [7–11]. This transcriptional plasticity may be key to allowing polyphagous arthropods to colonise diverse host plant species, but may also lead to host-dependent changes in their sensitivity to pesticides. For example, transfer of the generalist spider mite, *Tetranychus urticae* from an optimal host (bean) to a challenging host (tomato) resulted in whole-scale changes in gene expression and increased tolerance to three acaricides [12]. Work on whiteflies has also reported host plant effects on sensitivity to insecticides, with transfer of *Bemisia tabaci* and *T. vaporariorum* onto up to four different host plants resulting in significant differences in susceptibility to several insecticides used for control [13]. While these studies provide clear evidence that host plant origin can influence the sensitivity of whiteflies to synthetic insecticides the molecular basis of this, or if changes in susceptibility are associated with changes in gene expression, remain unknown.

As demonstrated by work on *T. urticae* [12] characterisation of the expression levels of all the genes in the genome of an insect when on different host plants can provide unique insights into the mechanisms underlying host-dependent changes in insecticide sensitivity. However, this approach is most effective when a fully annotated genome sequence is available as a reference. Previous work has sequenced, de novo assembled and annotated a reference transcriptome for *T. vaporariorum* [14]. This has provided an informative resource to identify enzyme families relevant to insecticide resistance and host-plant adaptation, however, many of the identified transcripts are partial, and the assembled transcriptome is unlikely to fully represent the complete gene content of *T. vaporariorum*. While the genomes of two different species of the *B. tabaci* species complex have been sequenced [15, 16], no genome currently exists for *T. vaporariorum.* Here we addressed this need by sequencing and annotating the draft genome of this species. We then leveraged this resource in combination with biological, transcriptomic and functional approaches to explore the relationship between host plant adaptation and insecticide sensitivity in *T. vaporariorum*. Five host plants were used in this study: *Cucumis sativus* (cucumber), *Nicotiana tabacum* (tobacco), *Cucurbita pepo* (pumpkin), *Phaseolus vulgaris* (French bean) and *Solanum lycopersicum* (tomato). Cucumber and pumpkin are representatives of the cucurbit family with tobacco and tomato representing the nightshades (Additional file 1: Figure S1). French bean divides the two families, represents an ‘ideal’ host and acted as a reference for comparative analyses. A variety of secondary metabolites are produced by these host plants. The Cucurbitaceae family produce bitter triterpenoid compounds called cucurbitacins that are toxic to many herbivores, with higher concentrations found in cucumbers than pumpkin [17]. Indeed, prior studies have shown increased carboxylesterase activity in whiteflies feeding on cucumber when compared to other plants [13]. The nightshade family, including tobacco and tomato, produce a variety of alkaloids, glycoalkaloids, terpenoids, organic acids and alcohols [18], the most notable nicotine – a potent natural insecticide. This makes them hostile host plants for most insect species.

## Results

### The genome of *T. vaporariorum*

Sequencing of a *T. vaporariorum* colony established from a single female using the 10X Genomics Chromium linked-read system generated 239 Gbp of sequencing data (Additional file 2: Table S1). k-mer analysis revealed a coverage peak at around 95X, and estimated a heterozygosity rate of 0.49% and a genome size of 591 Mbp (Additional file 3: Table S2 and Additional file 4: Figure S2A). The latter closely matches the genome size (615 MB) of the other sequenced whitefly species, *B. tabaci* [16]*.* Supernova effectively used 300 million raw short-reads with a minimum read length of 139.50 bp and molecule length of 33.75 kb (Additional file 5: Table S3) to generate a genome assembly of 581.92 Mb. The final assembly comprised 6016 scaffolds > 10 kb, with a contig N50 of 21.67 kb and scaffold N50 of 921.58 kb. The completeness of the gene space in the assembled genome was assessed using the Benchmarking Universal Single-Copy Orthologues (BUSCO) and Core Eukaryotic genes mapping approach (CEGMA) pipelines. BUSCO analysis identified 90.8, 92 and 93.5% of the Eukaryota, Insecta and Arthropoda test gene sets respectively as complete in the assembly (Additional file 4: Figure S2B). Furthermore, 94% of CEGMA core Eukaryotic genes (including both complete and partial genes) were present in the assembled genome (Additional file 6: Table S4). Structural genome annotation using a workflow incorporating RNAseq data predicted a total of 22,735 protein-coding genes (Additional file 7: Table S5). Of these 19,138 (79%) were successfully assigned functional annotation based on BLAST searches against the non-redundant protein database of NCBI and the InterPro database (Additional file 4: Figure S2C).

The proteome of *T. vaporariorum* was compared with *B. tabaci -v1.2, A. glabripennis -v2.0, T. castaneum -v5.2, M. persicae G006 -v1.0, A. pisum -v2.0 and D. melanogaster -v6.0* by orthology inference to obtain 15,881 gene clusters. Among these, 5345 gene clusters were found in all species of which 373 consisted entirely of single-copy genes. A total of 251 genes were specific to *T. vaporariorum*, 9841 genes were shared between *T. vaporariorum* and *B. tabaci*, and 7990, 7484, 8072, 7492 and 6805 genes are shared between *T. vaporariorum* and *A. glabripennis*, *T. castaneum*, *A. pisum*, *M. persicae* and *D. melanogaster* respectively. Based on *mcmctree* analysis, the divergence time between *T. vaporariorum* and *B. tabaci* was estimated to be approximately 110 million years ago (MYA).

**Fig. 1:**
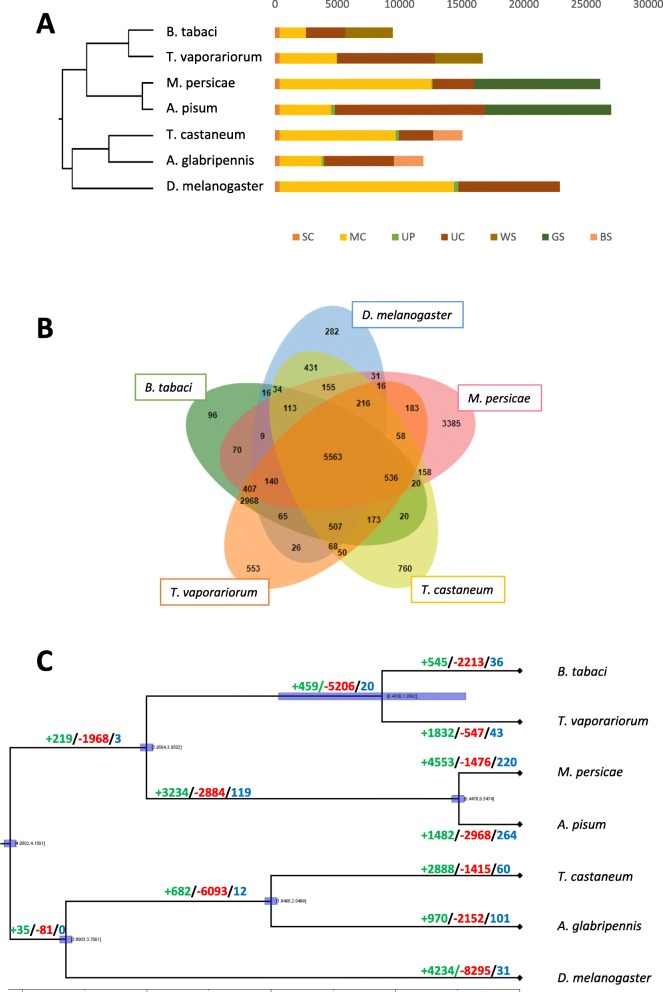
Phylogenomic analysis of *T. vaporariorum* and 6 other arthropod species. **a** Phylogenetic relationship and gene orthology of *T. vaporariorum* and other arthropods. SC indicates common orthologs with the same number of copies in different species, MC indicates common orthologs with different copy numbers in different species. UP indicates species-specific paralogs, UC indicates all genes which were not assigned to a gene family, WS, GS and BS indicate clade-specific genes. **b** Gene families shared by selected species. **c** Species dated phylogenetic tree and gene family evolution. Numbers on the branch indicate counts of gene families that are expanding (green), contracting (red) and rapidly evolving (blue). The horizontal blue bars correspond to 95% confidence intervals in time estimation based on the lognormal relaxed clock model

Modelling of global gene gain and loss revealed a gene turnover rate of 0.0026 gains and losses per gene per million years in *T. vaporariorum*, similar to that reported for *D. melanogaster* (0.0023 duplications/gene/million years) [19]. Estimation of gene gain and loss in gene families across the 7 arthropod species revealed a positive average gene family expansion (0.1427) in *T. vaporariorum*, with a greater number of gene families expanded (1832) and genes gained (2931) than contracted (587) or lost (734) (Additional file 8: Table S6). This contrasts with *B. tabaci* which has a negative (− 0.0993) average expansion resulting from a lower number of gene families expanded (545) and genes gained (1079) than contracted (2213) or lost (2600) (Additional file 8: Table S6). Thus, under the assumption of a constant gene gain and loss rate (ʎ) throughout the arthropod phylogeny, gene gain is higher and gene loss lower in *T. vaporariorum* than *B. tabaci* (Fig. 1c). Gene ontology (GO) enrichment analysis of genes specific to the whitefly clade, identified GO categories related to carbohydrate metabolism, peptidase activity, proteolysis and transferase activity as significantly enriched (*p* < 0.0001) (Additional file 9: Table S7). A total of 43 gene families were identified as rapidly evolving in *T. vaporariorum* with genes involved in metabolic processes, nucleic acid binding, and catalytic activity significantly enriched (Additional file 10: Table S8). Approximately 30% of the rapidly evolving genes gained in *T. vaporariorum*, are contracting in *B. tabaci* among which genes involved in transposase activity, DNA recombination, aspartic-type peptidase activity, actin filament binding, motor activity and cytoskeletal protein binding are significantly enriched.

### Curation and phylogeny of genes involved in detoxification of natural and synthetic xenobiotics

Because of our interests in the mechanisms underpinning adaptation of *T. vaporariorum* to plant secondary metabolites and insecticides we manually curated the gene superfamilies most frequently implicated in detoxification and/or excretion of these xenobiotics, namely cytochrome P450s (P450s), carboxyl/cholinesterases (CCEs), glutathione S-transferases (GSTs), UDP-glucuronosyltransferases (UGTs) and ATP-binding cassette transporters (ABC transporters) (Additional file 11: Table S9-S13). Phylogenetic analysis was then performed, with the curated gene sets of *T. vaporariorum* compared to those of *B. tabaci* (MEAM1) [16].

**Fig. 2:**
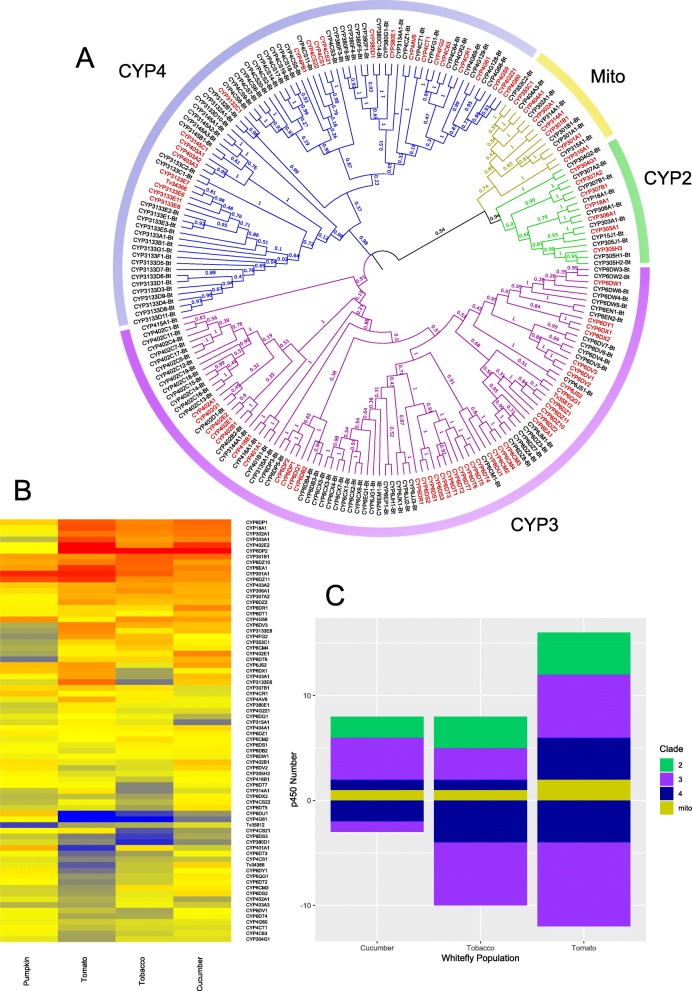
The CYPome of *T. vaporariorum*. **a** Maximum likelihood phylogeny of the CYPome of *B. tabaci* (black) and *T. vaporariorum* (red). Branches are coloured according to clade with bootstrap values from 1000 replicates given as decimals on branches. **b** Heatmap of log2 expression of full length *T. vaporariorum* cytochrome P450s in lines reared on tobacco (*Nicotiana tabacum*), tomato (*Solanum lycopersicum*), cucumber (*Cucumis sativus*) and pumpkin (*Cucurbita pepo*) when compared to a line reared on French bean (*Phaseolus vulgaris*). **c** Bar chart indicating numbers of P450s significantly over/under-expressed in the above populations. Bars are divided according to P450 clade

A total of 80 cytochrome P450s were identified in the *T. vaporariorum* genome assembly, representing an additional 23 novel genes beyond those previously described in the transcriptome of this species. While this takes the P450 gene count into the range of most other insect species (Additional file 12: Table S14), it is still significantly reduced when compared to *B. tabaci* which has 130 P450 genes. Phylogenetic comparison of the CYPome of *T. vaporariorum* and *B. tabaci* (Fig. 2a) revealed that both the CYP2 and mitochondrial clades are highly conserved between the two species with 1:1 orthologs observed for all members of the mitochondrial clan and only 3 additional enzymes found within the CYP2 clade of *B. tabaci*. However, significant differences in the CYPomes of the species are observed in the CYP3 and CYP4 clades. This is largely due to the presence or absence of certain P450 subfamiles in one of the species, or major expansions/contractions in other subfamilies. Within the CYP3 clan this is most apparent for the CYP402C (13 members in *B. tabaci* but none in *T. vaporariorum*), CYP6CX (7 members in *B. tabaci* but none in *T. vaporariorum*) and CYP6DT (no members in *B. tabaci* but 7 members in *T. vaporariorum*) subfamilies. While less marked than the above cases it is also notable that the CYP6CM subfamily comprises just one gene (*CYP6CM1*) in *B. tabaci* but three genes in *T. vaporariorum*. CYP6CM1 of *B. tabaci* is the most well characterised P450 in any whitefly species as its overexpression leads to resistance to several insecticides [20–23]. A similar pattern was observed in the CYP4 clade with the CYP3133 family, which is unique to the two whitefly species, comprising 19 genes and 7 subfamiles in *B. tabaci* but just one subfamily comprising 5 genes in *T. vaporariorum*. Likewise the CYP4CS subfamily contains 13 members in *B. tabaci* but only three members in *T. vaporariorum*. The net effect of the differences in the two clans sums to 17 additional CYP3 P450 genes and 31 CYP4 genes in *B. tabaci.* Both *T. vaporariorum* and *B. tabaci* are highly polyphagous so this disparity in P450 gene content is somewhat surprising, however, similar numbers of P450 genes are observed in the genomes of the generalist aphid *M. persicae* and the specialist *A. pisum* [24] demonstrating that CYPome size does not necessarily correlate with an insects host plant range.

In the case of GSTs a total of 26 genes were collated from the *T. vaporariorum* genome assembly - an addition of 4 sequences compared to the previous transcriptome. This number is comparable with other insect species and slightly higher than *B. tabaci* (24 genes). Interestingly, phylogeny (Additional file 13: Figure S3A) revealed a GST belonging to the epsilon class in *T. vaporariorum,* a clade not found in *B. tabaci* or indeed the sap sucking aphids *M. persicae* or *A. pisum* [25]. The largest clade in both whitefly species was the delta clan with 14 genes observed in *T. vaporariorum* and 12 in *B. tabaci*. Both the delta and epsilon classes of GSTs are unique to insects and members of this class have been previously implicated in detoxification of insecticides [26].

A total of 31 CCEs (4 novel) were identified in the *T. vaporariorum* genome. This is a comparable number to other insect species but again is reduced compared to *B. tabaci* which has 51 CCE genes. Phylogeny (Additional file 14: Figure S4A) assigned 14 of the *T. vaporariorum* CCE genes to the A and C clades, which have been previously associated with the detoxification of xenobiotics and metabolism of dietary compounds [27]. Despite the high number of CCEs in *B. tabaci* fewer of the CCE genes in this species are observed in these clades and so, with respect to xenobiotic tolerance, *T. vaporariorum* may be equally or even better equipped to hydrolyse allelochemicals and/or synthetic insecticides. *B. tabaci* has a larger total number of CCEs due to an expansion of CCEs belonging to the E clade which function to process hormones and pheromones [27]. Other clades principally related to neurodevelopment and cell adhesion remain largely consistent between the two whitefly species.

A total of 46 ABC transporters were curated from the *T. vaporariorum* genome, comparable with the number observed in *B. tabaci* (50) (Additional file 15: Figure S5A). In many of the clades (C, D, F and A) close to 1:1 orthology between the two species is observed. However, significant differences in the two species are observed in the B and G clades with many more ABC transporter genes observed in *B. tabaci* in the G clade and more genes in the B clade in *T. vaporariorum*. ABC transporters belonging to several clades (B, C, D and G) have been previously associated with detoxification of natural and synthetic xenobiotics in several arthropod species [28, 29]. These include *B. tabaci* where several ABC transporter genes of the G clade were implicated in resistance to neonicotinoids [30].

Comparison of the UGT gene family of *T. vaporariorum* with that previously described for *B. tabaci* [16] initially suggested that the genome of *B. tabaci* contains close to double the number of UGT genes (81) than the number observed in *T. vaporariorum* (42). However curation and naming (UGT nomenclature committee) of UGT genes in the two species revealed many of the previously proposed UGTs of *B. tabaci* were partial or not bona fide UGTs reducing the number in this species to 51 (Additional file 12: Table S14). Despite the similarity in UGT gene number in the two whitefly species, phylogenetic analysis (Additional file 16: Figure S6A) revealed marked contractions/expansions in specific UGT families between the two species. For example, the UGT353 family contained 1 gene in *T. vaporariorum* but 10 genes in *B. tabaci*. Such large species-specific blooms have been described in insect UGTs previously, for example, the UGT344 family of the pea aphid *A. pisum* and the UGT324, 325 and 326 families of red flour beetle (*Tribolium castaneum*) [31]. While other UGT families were observed in both *T. vaporariorum* and *B. tabaci* (UGT357, 358, 354), the pattern of one to one orthologs observed for several P450 subfamilies in the two species was not apparent (Additional file 16: Figure S6A). Previous analysis of insect UGTs [32] observed generally poor conservation between different insect species with genes frequently grouping in species-specific clades and our results are consistent with this. However, one clade that does not exhibit this pattern is the UGT50 family which is nearly universal across insect species, where it is composed of one member suggesting it has a conserved, and important, physiological role. Interestingly, while a single gene belonging to this family is found in *B. tabaci,* no member of this family was identified in *T. vaporariorum*, a phenomenon only previously reported for the pea aphid *A. pisum* [31].

In summary, across the five superfamilies of genes that play a key role in the ability of insects to detoxify and/or excrete natural and synthetic xenobiotics we observed ~ 1.4-fold difference in total gene number between *T. vaporariorum* (225) and *B. tabaci* (306). It has previously been suggested that species with larger complements of these families may be associated with a broader host range and greater propensity to develop resistance to chemical insecticides. However, both *T. vaporariorum* and *B. tabaci* are highly polyphagous and appear to be equally adept at evolving resistance to chemical insecticides [33]. Thus our findings support previous work which has found no direct link between host plant range, size of enzyme families and pesticide resistance [34, 35].

### Host-plant effects on the sensitivity of *T. vaporariorum* to insecticides

**Fig. 3:**
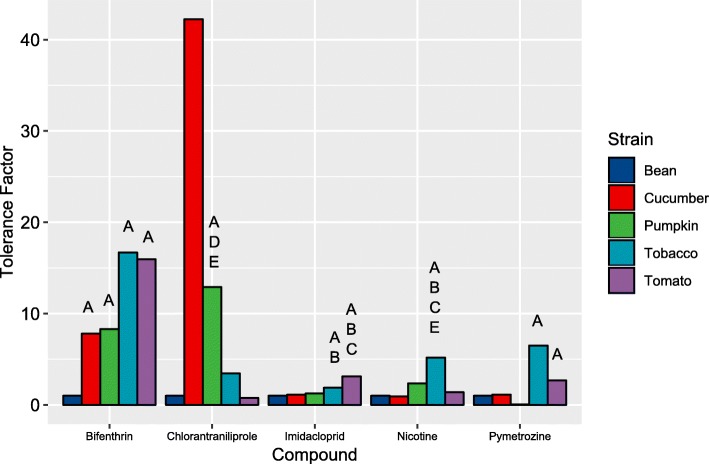
Relative sensitivity of five lines of *T. vaporariorum* reared on different host plants to five insecticides. Results are displayed as tolerance ratios relative to the line reared on French bean. Letters (A-E) are used to denote significant differences (A = significant to bean, B = cucumber, C = pumpkin, D = tobacco and E = tomato) based on non-overlapping 95% fiducial limits of LC_50_ values

To explore the relationship between the sensitivity of *T. vaporariorum* to natural or synthetic insecticides and the host plant on which it was reared we established cultures of the insecticide susceptible strain TV1 on bean, tobacco, tomato, cucumber and pumpkin. The sensitivity of each line to synthetic insecticides belonging to four different insecticide classes, and the plant secondary metabolite nicotine was then examined. The population reared on bean, the host of origin, acted as a reference for the calculation of tolerance ratios (TRs). Adaptation to different host-plants was frequently associated with significant decreases in sensitivity to insecticides (Fig. 3, Additional file 17: Table S15). This was particularly apparent for the nightshade hosts (tobacco and tomato) which in general exhibited a higher tolerance to the tested insecticides than all other lines. All lines showed significant tolerance to the pyrethroid bifenthrin compared to the line on bean and this was particularly pronounced for the tobacco and tomato lines (TRs of 16 in both cases). Similarly, the lines reared on tobacco and tomato show significant tolerance to the antifeedant pymetrozine and the neonicotinoid imidacloprid compared to the bean-reared line. However, the most dramatic changes in sensitivity were observed for the diamide chlorantraniliprole. In this case the cucurbits, in particular cucumber, showed marked tolerance to this compound compared to both the bean-reared (TR of 42) and nightshade-reared lines (TR of 12–55). In the case of the natural insecticide nicotine only the tobacco-reared line exhibited a significant reduction in tolerance to this compound.

These data, in combination with a range of previous studies (see introduction), demonstrate unequivocally that host plant can strongly influence the susceptibility of herbivorous insects to insecticides. It is notable that the *T. vaporariorum* lines reared on the nightshade hosts showed the broadest spectrum of tolerance to the tested insecticides. Tobacco and tomato are challenging hosts for most insect species due to the profile of insecticidal allelochemicals they produce (see introduction). This finding is therefore consistent with previous studies [12, 36–45] which have provided strong evidence that host-dependent insecticide tolerance results, in part, from induction of insect detoxification pathways in response to plant allelochemicals.

### Host-plant effects on *T. vaporariorum* gene expression

**Fig. 4:**
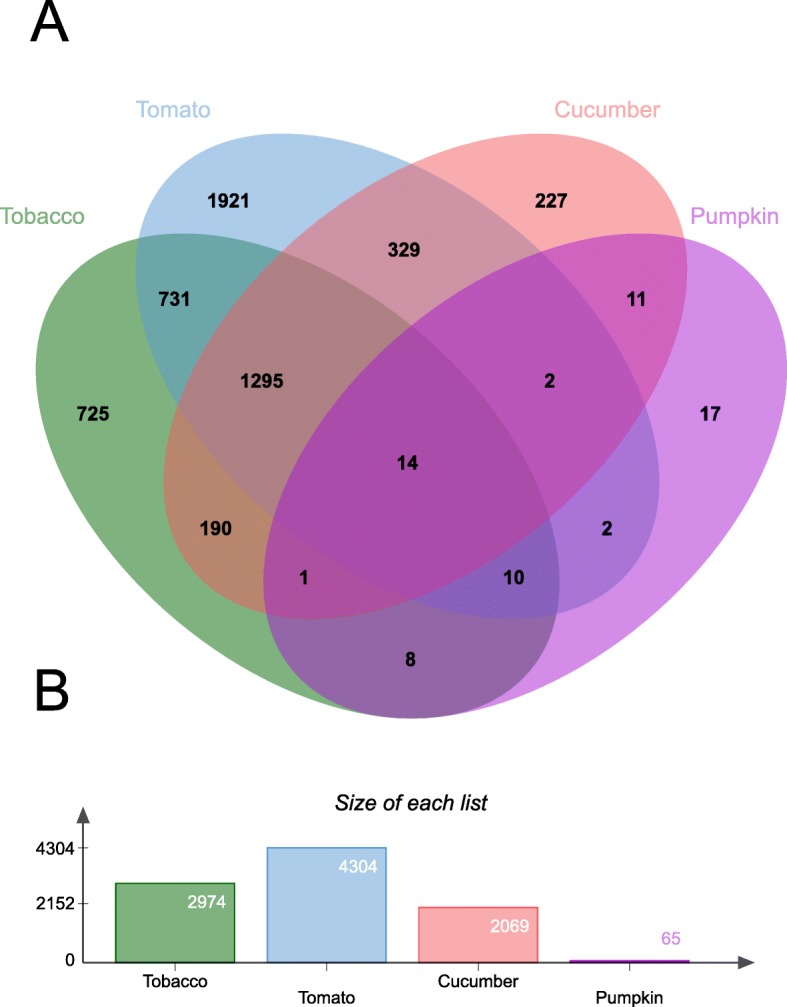
The transcriptional response of *T. vaporariorum* during adaptation to different host plants. **a** Venn diagram showing numbers of differentially expressed genes between *T. vaporariorum* lines reared on tobacco (*Nicotiana tabacum*), tomato (*Solanum lycopersicum*), cucumber (*Cucumis sativus*) and pumpkin (*Cucurbita pepo*). **b** Bar charts indicate total number of genes differentially expressed in each RNAseq comparison. All lines were compared to a reference line of *T. vaporariorum* reared on French bean (*Phaseolus vulgaris*)

To examine if changes in insecticide sensitivity of the host-adapted lines were correlated with changes in gene expression we performed replicated messenger RNA sequencing (RNAseq) of each *T. vaporariorum* line. Comparisons against the bean-reared line identified 65–4304 significantly differentially expressed (DE) genes (Fig. 4b, Additional file 18: Tables S16-S19), with a greater number of genes upregulated in lines reared on the alternate (non-bean) host plant. The most dramatic transcriptional response was observed for the nightshade-reared lines with 4304 and 2974 genes identified as DE in the tomato and tobacco-reared lines compared to the control line on bean. In contrast, just 65 genes were DE between the pumpkin- and bean-reared *T. vaporariorum* lines, with an intermediate number of genes (2069) DE in the comparison with the cucumber-reared line. Comparison of the lists of DE genes revealed clear plant-family specific transcriptional signatures with the nightshade derived lines sharing more DE genes with each other than with either of the cucurbit-reared lines and vice versa (Fig. 4a). This clear evidence of a plant-specific transcriptional response has also been observed in Lepidoptera and spider mites [9, 11, 12]. The magnitude of the transcriptional response of *T. vaporariorum* to the different host plants is consistent with the profile of the defensive secondary metabolites they produce. Our results suggest extensive transcriptional reprogramming is required for *T. vaporariorum* to effectively utilise the nightshades as hosts, which produce a challenging profile of allelochemicals including potent natural insecticides. In contrast, our data suggest that only a limited transcriptional response is required for *T. vaporariorum* to adapt from bean to pumpkin, which produces a lower concentration of the anti-herbivore cucurbitacins than cucumber - on which *T. vaporariorum* exhibited more extensive remodelling of gene expression. Thus, generalism in *T. vaporariorum* is associated with marked transcriptional plasticity. This finding provides further vidence that polyphagous species can rapidly tailor gene expression for a particular host and this plasticity plays an important role in their striking ability to utilise a diverse range of plants.

Gene ontology (GO)-term enrichment analysis identified significantly enriched processes for both the tobacco-reared and the tomato-reared comparisons, however, no over- or under-represented terms were identified in the RNAseq comparisons involving the cucumber or pumpkin-reared lines (Additional file 19: Figure S7). The significantly enriched terms for the tomato-reared comparison primarily relate to nucleic acids with many of the terms involving nucleotide, nucleoside and ribonucleotide binding. This likely reflects the DE of genes involved in regulating the large scale transcriptional changes observed in the tomato-reared comparison (see below) and parallels the findings of previous research on host-plant adaptation of the polyphagous butterfly, *Polygonia c-album* [9]. Interestingly, the same terms were enriched in the genes classed as rapidly evolving in *T. vaporariorum* (see above). The majority of the enriched terms in the tobacco-reared comparison reflect metabolic processes and ranged from higher-level terms such as primary metabolism to more specific terms such as heterocyclic compound and nitrogen compound metabolism. In regards to the two latter terms it is notable that nicotine, the natural insecticide produced by tobacco, is a heterocyclic nitrogen compound. Finally, the list of enriched terms also included ‘catalytic activity’ which is synonymous with enhanced enzyme activity, and may reflect a response to the allelochemicals produced by tobacco. The only significantly enriched term shared by the tobacco-reared and tomato-reared comparisons was ‘ion binding’.

QPCR was used to validate the expression of 6 genes selected randomly from those that were DE across RNAseq comparisons, and three P450s *CYP6CM2*, *CYP6CM3* and *CYP6CM4* that show high similarity to a known insecticide resistance gene (*CYP6CM1*) in *B. tabaci*. All genes were validated as DE although the fold-changes observed in QPCR were lower than those reported by edgeR in RNAseq analysis (Additional file 20: Figure S8).

### Detoxification and transport of natural and synthetic xenobiotics

To build on our earlier analysis of genes involved in the detoxification and/or excretion of natural and synthetic insecticides we examined the expression of genes encoding P450s, GSTs, CCEs, UGTs and ABC transporters, and/or also interrogated lists of DE for genes encoding these proteins (Additional file 21: Table S22). Analysis of candidate genes focused on the tobacco-, tomato- and cucumber-reared *T. vaporariorum* lines, which exhibited the greatest transcriptional response, and exploring the association between upregulation of detoxification genes and sensitivity to insecticides.

Of all detoxification enzyme superfamilies P450s have been most frequently implicated in tolerance to plant allelochemicals and synthetic insecticides [46], and, in a previous study on spider mites, showed the most profound changes in gene expression after transfer to a challenging host [12]. Consistent with these studies we observed marked differences in the expression of P450 genes between the whitefly lines adapted to novel host plants (Fig. 2b, Additional file 18: Tables S16-S21). Interestingly, the lines with the most similar profile of P450 expression were the cucumber and tobacco-reared lines (Fig. 2b). The expression profile of the pumpkin-reared line was more distantly related to that of the other three strains and also had no significantly over-expressed P450s relative to the bean-reared line. A total of 11, 18 and 28 P450 genes were DE in the cucumber-, tobacco- and tomato-reared *T. vaporariorum* lines respectively. Grouping these by clade (Fig. 2c) revealed the majority belong to the CYP3 and 4 clades, members of which have been most frequently linked to xenobiotic detoxification across a range of insect species. Five P450 genes were overexpressed in all three comparisons of which *CYP6DP2* belonging to the CYP3 clade was by far the most highly expressed in all three lines (19.6–28.3-fold) (Fig. 2b). Two additional P450s were over-expressed in both lines reared on nightshade hosts; *CYP6EA1* a member of the CYP3 clade (overexpressed 5.0–9.2-fold) and *CYP306A1* (overexpressed 3.3–2.4-fold). Finally, as detailed above, QPCR revealed that three P450s, *CYP6CM2*, *CYP6CM3* and *CYP6CM4*, were overexpressed in the tobacco-reared line (2.4–4.7-fold) that belong to the same subfamily as *CYP6CM1* of *B. tabaci* (Additional file 20: Figure S8). The overexpression of *CYP6CM1* in this species has been shown to confer potent resistance to several neonicotinoid insecticides which have structural similarity to nicotine [21, 23]. Correlation of the expression of the upregulated P450s with the phenotypic data derived from insecticide bioassays allowed us to assess their potential role in mediating the observed tolerance of the different *T. vaporariorum* lines to insecticides. While *CYP6DP2* is the most highly upregulated P450 in the cucumber-, tobacco- and tomato- reared lines, correlation of its expression with bioassay data suggests it may play a limited role in insecticide tolerance. Specifically, this P450 is overexpressed > 20-fold in the cucumber-reared line but is not overexpressed in the pumpkin reared line, despite this both of these lines show the same (~ 8-fold) tolerance to bifenthrin (Fig. 3), suggesting its overexpression has no effect on the sensitivity of *T. vaporariorum* to this compound. Similarly, the cucumber-reared line exhibits no tolerance to imidacloprid, pymetrozine or nicotine (Fig. 3), suggesting the overexpression of *CYP6DP2* does not enhance the detoxification of these compounds. Finally, the high expression of *CYP6DP2* in the tomato-reared line is not associated with tolerance to chlorantraniliprole (Fig. 3). Thus, the overexpression of this P450 in three of the lines may represent a generic stress response to challenging host plants, but is unlikely to explain the pattern of insecticide tolerance observed. Using the same process all other overexpressed P450s were ruled out as strong candidate insecticide tolerance genes except for *CYP6EA1*. This P450 is overexpressed in the tobacco and tomato- reared lines and is a candidate for the tolerance of these lines to imidacloprid, with the level of expression in the two lines (5.0-fold and 9.2-fold) mirroring their relative tolerance to this compound (3.1-fold and 5.2-fold). Finally, given previous work on the substrate profile of CYP6CM1 in *B. tabaci*, the overexpression of *CYP6CM2–4* in the tobacco-reared line represent potential candidates to explain the tolerance of this line to nicotine (Fig. 3).

In the case of GSTs two genes were upregulated in the cucumber-reared line (g10036 and g13867), however, both of these were also overexpressed at similar levels in both night-shade reared lines (Additional file 13: Figure S3B and Additional file 18: Tables S16, S20). This suggests that while they may play a role in host plant adaptation they play no role in the enhanced tolerance of the cucumber-reared line to chlorantraniliprole, or the tolerance of the nightshade-reared lines to pymetrozine or imidacloprid (Fig. 3). In addition to these two genes, one further GST (g5077) was upregulated exclusively in the nightshade-reared plants (overexpressed 2.7- and 2.3-fold in the tobacco- and tomato-reared lines) (Additional file 18: Table S20). This GST belongs to the microsomal clade and while its pattern of expression in the two nightshade reared lines would make it a candidate for contributing to the observed tolerance of these lines to bifenthrin (Fig. 3), to date, only cytosolic GSTs have ever been implicated in insecticide resistance [47]. No additional GSTs were overexpressed exclusively (or at significantly higher levels) in the tobacco-reared lines that might contribute to the tolerance of this line to nicotine.

Two CCEs, g14105 and g17172, were upregulated in the cucumber-reared line, of which the latter was also modestly overexpressed in the nightshade-reared lines (Additional file 14: Figure S4B and Additional file 18: Table S16, S20). The high expression of g14105 (11.9-fold overexpressed) and the fact that it belongs to clade A, members of which have been previously associated with the detoxification of xenobiotics and metabolism of dietary compounds [27], makes it a potential candidate for the tolerance of the cucumber-reared line to chlorantraniliprole (Fig. 3). g17172 also belongs to clade A, however, comparison of its pattern of expression in the three *T. vaporariorum* lines with the sensitivity of these lines to insecticides suggests it is unlikely to confer tolerance to any of the compounds tested.

Much more marked changes were observed in the expression of genes encoding UGTs, with 11 UGT genes upregulated in the cucumber-reared line and 9 upregulated in both nightshade-reared plants (Additional file 16: Figure S6B and Additional file 18: Table S16, S20). Of these 7 were upregulated at similar levels in all three lines. The four UGT genes (*UGT352P5*, *UGT356E1*, *UGT352P2* and *UGT358B1*) exclusively upregulated (2.3–4.5-fold) in the cucumber-reared line are potential candidates for a role in the marked tolerance of this line to chlorantraniliprole. Indeed, UGTs have been recently implicated in metabolic resistance to this compound in the diamondback moth, *Plutella xylostella*, and striped rice stem borer, *Chilo suppressalis* [48, 49]. The two UGTs (g12287 and g2864) exclusively overexpressed in the nightshade reared lines are potential candidate genes for a role in the tolerance of these lines to insecticides, particularly g12287 which was overexpressed > 19-fold in both lines.

Several ABC transporters were found to be significantly overexpressed in response to feeding on cucumber, tobacco and tomato, although few were upregulated to the extent seen for other families of detoxification genes (Additional file 15: Figure S5B and Additional file 18: Tables S16, S18, S19). Four genes (g11125, g11231, g5414 and g3563) were moderately (up to 5.4-fold) overexpressed in the cucumber-feeding line. ABC transporter genes have previously been implicated in insecticide resistance in *B. tabaci,* all belonging to the G clade [30]. Three of the ABC transporter genes overexpressed in the cucumber-reared line (g11231, g5414 and g3563) also belong to this clade and thus are potential candidates for the increased tolerance to chlorantraniliprole. Both genes significantly upregulated in the tobacco-reared line (g11231 and g5415) were also upregulated in the tomato-reared line, and so are unlikely to be responsible for the tolerance of this line to nicotine (Fig. 3). However, they could be associated with the elevated tolerance to imidacloprid or pymetrozine, especially as ABC transporters belonging to the G clade have been associated with neonicotinoid resistance in *B. tabaci* [30].

### Structural proteins and cysteine proteases

Analysis of the transcriptomes of the *T. vaporariorum* lines revealed other trends in the transcriptional response to host switching beyond changes in the expression of genes belonging to superfamilies commonly implicated in detoxification. These included marked changes in the expression of genes encoding cathepsin B cysteine proteases and cuticular proteins, both of which have been previously implicated in insect adjustment to new host plants [24]. In the case of cathepsin B proteases the tomato, tobacco and cucumber reared lines all had > 10 genes belonging to this family DE (Additional file 18: Tables S16, S18, S19). In the cucumber-reared line all but one of the 14 cathepsin B genes DE was upregulated (2.1- to 14.6-fold), however, in both the tobacco and tomato reared lines a higher number of cathepsin B genes were downregulated with just 3 genes upregulated (2.7- to 30.2-fold) in both comparisons (Additional file 18: Table S18). Previous work on the aphid, *M. persicae* identified marked downregulation of cathepsin B genes in aphids when transferred from cabbage (*Brassica rapa*) to *Nicotiana benthamiana*, a close relative of tobacco [24]. RNAi-mediated knock-down of genes belonging to this family impacted aphid fitness in a host-dependant manner providing clear evidence that cathepsin B genes play a role in adaptation to specific host plants [24]. Cathepsin B proteins have a role in several biological processes in insects including digestion, embryonic development, metamorphosis and the decomposition of larvae and adult fat body. Their specific role in host plant adaptation in less clear but their overexpression could represent a counter defence against plant protease inhibitors [50]. Alternatively, work on aphids has suggested they may function as effectors that manipulate plant cell processes in order to promote insect virulence [24].

In the case of genes encoding structural components of the insect cuticle 15 sequences were identified as over-expressed in the nightshade-reared *T. vaporariorum* lines that returned BLAST hits to cuticle proteins and cuticular protein precursors (Additional file 18: Table S20). All proteins which were characterised belonged to the Rebers and Riddiford subgroup 2 (RR-2) cuticular family and so are associated with hard cuticle rather than flexible cuticle [51]. These findings align with prior studies on *M. persicae*, *Polygonia c-album* and *B. tabaci* which all reported the upregulation of genes encoding cuticular proteins during host adaptation [9, 12, 52]. The specific role of cuticular proteins in insect host plant adaptation is unclear, however, a study of the adaptation of *B. tabaci* to tobacco observed both the upregulation of cuticular proteins and increases in body volume and muscle content [52]. Thus, the overexpression of cuticular proteins could play a role in host plant adaptation by mediating physical changes that allow insects to more readily survive the effects of feeding on hostile plants, and this in turn could impact their sensitivity to insecticides.

### Gene regulation and signalling

Among the most striking changes in gene expression during host adaptation related to genes involved in the regulation of transcription and signal transduction namely transcription factors and G protein-coupled receptors (GPCRs).

Transcription factors have been shown to play a key role in the regulation of enzymes responsible for detoxifying xenobiotics [53–56]. Their potential role in underpinning the marked transcriptional response observed during the adaptation of *T. vaporariorum* to challenging host plants was suggested by the over-expression of 56 transcription factors in the tomato- and tobacco-reared lines, representing 5.1% of all DE genes (Additional file 18: Table S20). The overexpressed genes encoded factors belonging to a variety of families including zinc-finger (ZF-TFs) and nuclear hormone receptors (NHR). ZF-TFs have been previously associated with the regulation of a ribosomal protein associated with pyrethroid resistance in mosquitoes [57], and a transcription factor belonging to the NHR family was upregulated in *T. urticae* in response to transfer to tomato and in two insecticide resistant strains [12]. However, it is worth noting that many of the observed changes in the expression of transcription factors may be unrelated to hostile challenge or insecticide resistance but simply result from the change in the nutrient composition of the host plant.

G-protein-coupled receptors or GPCRs are the largest family of membrane proteins, responsible for cellular responses to hormones and neurotransmitters [58]. More than 20 genes annotated as GPCRs were overexpressed during adaptation of *T. vaporariorum* to nightshade plants (Additional file 18: Table S18). The stress of feeding on these challenging plants could lead to upregulation of these proteins for several reasons. Firstly, GPCRs mediate neurohormones which have been implicated in the regulation of feeding and digestion in insects which are likely modified when feeding on hostile plants [59–61]. Secondly, previous work in mosquitoes found that knocking out GPCR genes not only reduces insecticide resistance but also downregulates the expression of P450 genes, suggesting a role for GPCRs in the regulation of these enzymes [62]. As the significant upregulation of GPCRs in the nightshade-reared lines was associated with both induced tolerance to insecticides and significant over-expression of P450s, it is possible that GPCRs play a similar role here.

### P450s of the CYP6CM1 subfamily confer tolerance to plant-derived, but not synthetic, insecticides

**Fig. 5:**
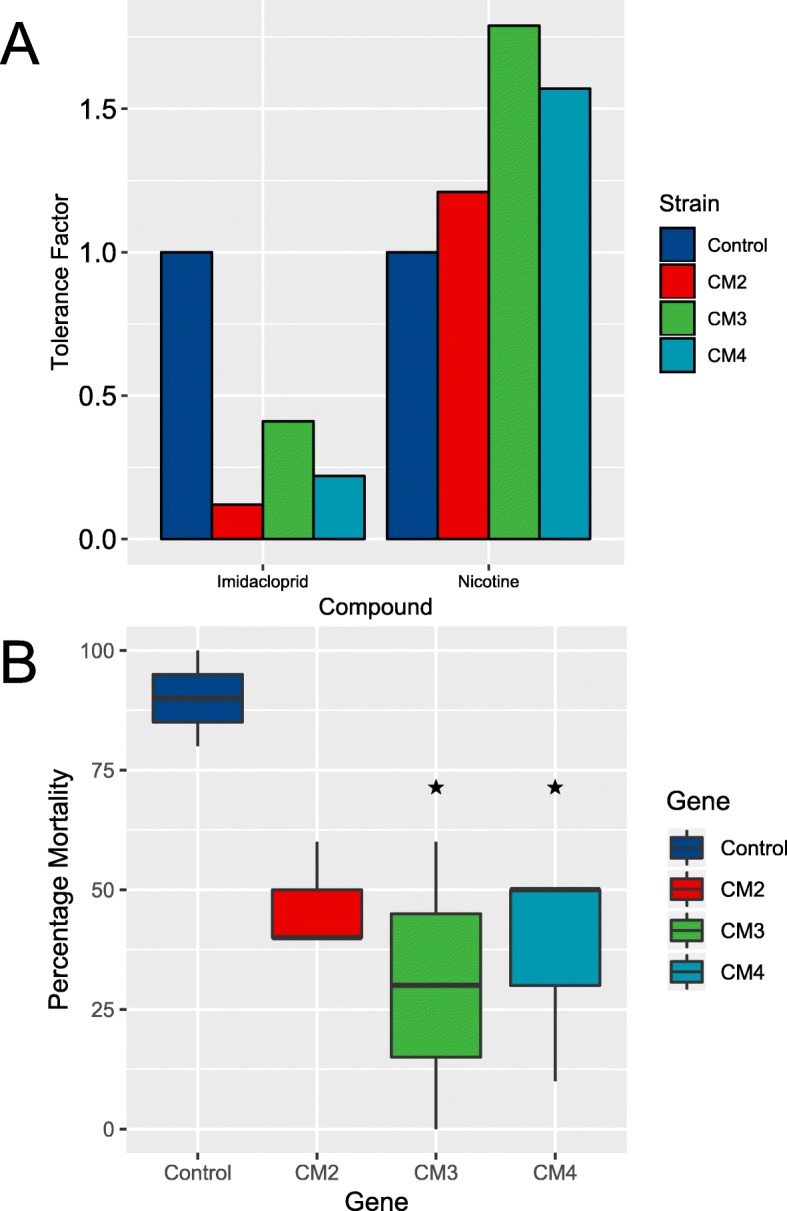
Sensitivity of transgenic strains of *D. melanogaster* expressing the *T. vaporariorum* P450s *CYP6CM2*, *CYP6CM3* or *CYP6CM4* to two insecticides. **a** Tolerance factors of three transgenic *D. melanogaster* strains expressing *CYP6CM2*, *3* or *4*, to nicotine and imidacloprid compared to a control line (flies of the same genetic background but without the transgene). **b** Percentage mortality of the same strains when exposed to a single high concentration (30,000 ppm) of nicotine. Stars indicate significance relative to the control strain, calculated by a one-way ANOVA and post-hoc Tukey test (*P* < 0.05)

As described above transcriptome profiling identified a diverse range of candidate insecticide tolerance genes which require functional characterisation to confirm their causal role. As a first step towards this aim we selected P450s of the CYP6CM subfamily for further functional characterisation for the following reasons: Firstly, the three P450s belonging to this subfamily in *T. vaporariorum* were all overexpressed in the tobacco-reared line which exhibited tolerance to both nicotine and imidacloprid (Additional file 20: Figure S8). Secondly, in a previous study two of the genes, *CYP6CM2* and *CYP6CM3*, were found to be upregulated in imidacloprid-resistant populations of *T. vaporariorum* from Greece [21, 23]. Finally, the three P450s belong to the same subfamily as CYP6CM1, a P450 in *B. tabaci* that confers strong resistance to several neonicotinoid insecticides including imidacloprid [23]. CYP6CM2–4 thus represent strong candidates for P450 enzymes that confer resistance to a natural insecticide (nicotine) and a structurally related synthetic insecticide (imidacloprid). To investigate this transgenic strains of *D. melanogaster* were created that individually express each of the three genes, and their sensitivity to nicotine and neonicotinoids examined. In insecticide bioassays none of the three lines showed tolerance to the neonicotinoid imidacloprid (Fig. 5a, Additional file 22: Table S23). Indeed, all three lines were much more sensitive to this compound than flies of the same genetic background but without a transgene, suggesting a fitness cost is associated with the expression of these transgenes in *D. melanogaster*. In contrast, in bioassays with nicotine a trend of increased tolerance of the three transgenic lines to this compound was observed when compared to the control. While the 95% confidence intervals of the calculated LC_50_ values between control and transgene expressing lines overlap, the lines expressing *CYP6CM3* and *CYP6CM4* both showed significant resistance compared to the control when exposed to a 30,000 ppm concentration of nicotine (one-way ANOVA, *p* < 0.05, post hoc: Control-CM3 and Control-CM4 *p* < 0.05). These data provide evidence that these P450s confer tolerance to nicotine but not to synthetic insecticides. The latter finding is consistent with a recent study which expressed CYP6CM2 and CYP6CM3 in *E. coli* and observed no metabolism of the neonicotinoid insecticides imidacloprid, clothianidin, dinotefuran, thiamethoxam, nitenpyram, thiacloprid, or acetamiprid [63].

## Discussion

The genome sequence of *T. vaporariorum* reported here represents a valuable resource for future research on this important agricultural pest. Comparison of gene superfamilies most commonly involved in the detoxification, transport and excretion of xenobiotics with those of the tobacco whitefly *B. tabaci* revealed a marked difference in gene complement between the two species in many of these families. This finding was unexpected given the fact that both species belong to the Aleyrodidae family, are highly polyphagous and equally adept at evolving resistance to chemical insecticides. Furthermore, it provides additional evidence that the number of detoxification genes per se does not define the capacity of insect species to tolerate (or evolve tolerance to) toxic xenobiotics.

Leveraging the new genomic resource to explore the molecular basis of host plant adaptation in *T. vaporariorum* revealed that polyphagy in this species is associated with marked transcriptional plasticity. This aligns with the results of studies on host adaptation in several other insect species which, in combination, provide strong evidence that generalist species rapidly modulate gene expression in a host-dependent manner. Thus transcriptional plasticity likely plays a key role in the striking ability of such species to utilise a diverse range of plants as hosts. The mechanisms underpinning the observed changes in gene expression during the adjustment of generalist insects to new host plants are currently less clear and could result from induction via signalling pathways, selection on standing genetic variation in the population (in sexual species), and/or epigenetic regulation of gene expression. Our results suggest that in *T. vaporariorum* induction via signal transduction pathways leading to the modulation of transcription factor expression may be an important driver of the transcriptional response observed.

Importantly, our data reveal that the observed reprogramming of gene expression during whitefly host adaptation is associated with marked changes in tolerance to natural and synthetic insecticides. Several genes encoding enzymes or transporters that belong to families or clades previously implicated in the detoxification of xenobiotics were upregulated in the insecticide tolerant lines and represent candidate tolerance genes for further analysis. We demonstrate one route for the characterisation of such genes by expressing *T. vaporariorum CYP6CM2–4* in *D. melanogaster*, and provide evidence that two of these P450s confer tolerance to nicotine but not to synthetic neonicotinoid insecticides.

Besides providing insights into the molecular basis of polyphagy and host plant adaptation in *T. vaporariorum* the results of our study may also have applied implications for control of this species using insecticides. We find that the sensitivity of *T. vaporariorum* to a specific insecticide can vary by more than 40-fold depending on the host plant on which it is feeding. The practical relevance of these host-dependent differences in insecticide sensitivity for control is unknown and requires further research to establish. However, our findings highlight the importance of assessing the sensitivity of *T. vaporariorum* to insecticides directly on the target crop in order to define appropriate label rates. This may be especially relevant in the context of off-label insecticide use where there may be limited background research on the appropriate application of an insecticide in the novel setting.

## Conclusions

We present the first reference genome sequence for *T. vaporariorum* and demonstrate the utility of this resource in enabling whole genome and transcriptome analyses. Our findings provide further evidence of the ability of polyphagous insects to extensively reprogram gene expression during host adaptation and illustrate the potential implications of this on their sensitivity to synthetic insecticides.

## Methods

### Insect strains

The TV1 strain of *T. vaporariorum* was used for all experiments described in this study. This is a long-term laboratory culture that is susceptible to insecticides, and is normally reared on French bean (*Phaseolus vulgaris* L., cv. ‘Canadian Wonder’). All cultures of TV1 described in this study were reared at 24 °C, 55% relative humidity, with a 16/8 h (day/night) light cycle.

### Sequencing, assembly and annotation of the *T. vaporariorum* genome

In order to reduce the heterozygosity of the TV1 strain prior to sequencing a single female adult whitefly was placed on French bean to oviposit and establish a colony. Four hundred fifty mixed sex adults of the resulting colony were removed for DNA extraction. High molecular weight genomic DNA was extracted using the Genomic-tip 20/g kit (Qiagen) according to the manufacturer’s instructions and eluted in tris-EDTA buffer.

Chromium 10x libraries were prepared from genomic DNA and sequenced on a single Illumina HiSeq lane using a 2x150bp paired end configuration by GeneWiz (South Plainfield, New Jersey, USA) to generate > 350 M raw paired-end reads. Genome characteristics were estimated using short read data and a k-mer based approach implemented in GenomeScope [64]. Illumina reads were quality filtered and subjected to 19-mer frequency distribution analysis applying Jellyfish –v2.2.10 [65]. Raw reads were assembled using Supernova -version 2.0.0 [66] with multiple assemblies performed using different parameter settings. The best results were obtained with the parameter *-maxreads* value of 257,600,000. The completeness of the gene space in the assembled genome was assessed by BUSCO (Benchmarking universal single-copy orthologs) –v3.0.2 [67] and CEGMA (Core Eukaryotic genes mapping approach) –v2.5.0 [68] analysis.

Prior to gene prediction, the genome of *T. vaporariorum* was masked for repetitive elements using RepeatMasker –v4.0.7 [69]. RepeatModeler –v1.0.11 [70] was also used to identify repetitive sequences and transposable elements. Repeats originating from coding regions were removed by performing a BLAST search against the proteome of *B. tabaci* with sequences returning hits at e-value >1e-10 filtered out. The RepBase –v24.05 [71] library was then merged with the repeats predicted by RepeatModeler and used to mask the *T. vaporariorum* genome. Protein coding genes were predicted using GeneMark-ES –v4.3.8 [72] and AUGUSTUS –v3.3.0 [73] implemented in the BRAKER -2.1.2 [74] pipeline using RNA-seq alignments as evidence. *T. vaporariorum* RNA-seq datasets (see below) were individually mapped against the repeat masked genome using STAR –v2.7.1 [75]. The bam files from individual samples were then combined and fed into BRAKER. Low quality genes consisting of fewer than 50 amino acids and/or exhibiting premature termination were removed from the final gene set. Functional annotation of the *de-novo* predicted gene models was performed based on homology searches against the NCBI nr and Interpro databases using BLAST2GO –v5.2.5.

### Ortholog analysis

To characterize orthology and compare gene family evolution to other insects the final annotation set for *T. vaporariorum* was compared to 6 other arthropod genomes. The proteomes of *B. tabaci -v1.2, Anoplophora glabripennis -v2.0, Tribolium castaneum -v5.2, Myzus persicae G006 -v1.0, Acyrthosiphon pisum -v2.0 and Drosophila melanogaster -v6.0* were used to define orthologous groups of genes (gene families) between these peptide sets using OrthoFinder -v1.1.8 [76].

### Species level phylogeny and divergence time estimation

Phylogenetic analysis was performed using single-copy orthologous genes from common gene families identified by OrthoFinder. A species tree was also generated using the OrthoFinder pipeline with *D. melanogaster* used as an outgroup. The species tree was rooted using the STRIDE –v1.0.0 [77] algorithm within OrthoFinder. MCMCTREE, as implemented in PAML v4.9e [78], was then used to estimate the divergence times of *T. vaporariorum* by approximate likelihood calculation. For this, substitution rate was estimated using codeml by applying root divergence age between Diptera, Coleoptera and Hymenoptera as 410 MY. This is a simple fossil calibration for the most common recent ancestor of the three families. The estimated substitution rate (0.107532) was the per site substitution rate for the amino acid data set and used to set priors for the mean substitution rate in Bayesian analysis. As a second step, gradient and hessian of branch lengths for all 7 species were also estimated. Finally, the tree file with fossil calibrations, the gradient vector and hessian matrices file and the concatenated gene alignment information were used in the approximate likelihood calculation. The parameter settings of MCMCTREE were as follows: clock = 2, model = 3, BDparas = 110, kappa_gamma = 6 2, alpha_gamma = 11, rgene_gamma = 9.3, and sigma2_gamma = 1 4.5.

### Analysis of gene family evolution

Gene family evolution across the arthropods described above was investigated using CAFE -v.3.0.0 [79]. The matrix of gene family sizes, as obtained from OrthoFinder, was used as input in CAFE and the evolution of gene families modelled along the dated species tree.

### Curation of candidate gene families

Contigs were initially run through the NCBI Blastx remote server to discover sequences with homology to the gene family of interest using an E-value cut off value of 1e-5. Contigs returning relevant hits were then manually curated using Geneious software v9.0.5 (Biomatters Ltd., Auckland, New Zealand). Contigs were also compared with the previously assembled transcriptome of *T. vaporariorum* [14] and any previously assigned nomenclature transferred as appropriate. Genes encoding novel P450s were named by Dr. David Nelson in accordance with the conventions of the P450 nomenclature committee (http://drnelson.uthsc.edu/cytochromeP450.html) [80]. Similarly, UDP-glucuronosyltransferases UGTs were named by Dr. Michael Court in accordance with the conventions of the UGT nomenclature committee (http://prime.vetmed.wsu.edu/resources/udp-glucuronsyltransferase-homepage/ugt-submission-instructions) [81].

### Gene level phylogeny

Sequences were imported into MEGA X [82] which was used to perform multiple sequence alignments for each family of genes using MUSCLE. The same software was also utilised to determine the most reliable substitution and rate variation model for further phylogenetic analysis. Gene sets for each relevant enzyme family were obtained from *B. tabaci* (MEAM1), and when necessary *A. pisum*, in order to more reliably assign *T. vaporariorum* genes of interest into clades. Phylogenetic trees were then created in MEGA from the aligned sequences using a maximum likelihood model with a bootstrap value of 1000.

### Whitefly bioassays

Colonies of *T. vaporariorum* (Tv1) were established on 5 different host plants; tobacco (*N. tabacum*), tomato (*S. lycopersicum*), pumpkin (*C. pepo*), cucumber (*C. sativus*) and French bean (*P. vulgaris*). All colonies were allowed to establish for > 7 generations before bioassays took place. In order to avoid potential confounding effects from performing bioassays directly on host plants whiteflies were exposed to insecticide by artificial feeding. Insecticides were initially dissolved in acetone and then diluted in a 15% sucrose solution. Three hundred uL of each insecticide was then applied to 55 mm petri dishes between two stretched pieces of parafilm to make a feeding sachet as described previously [83]. Whiteflies were removed from each host plant and anaesthetised using carbon dioxide. Twenty adults of mixed sex were added to each petri dish with each concentration tested in triplicate for each host plant. Mortality was then recorded according to IRAC guidelines for each pesticide [84]. Probit analysis was used to calculate LC_50_ values and 95% confidence intervals (PoloPlus, LeOra Software Company).

### RNA sequencing

RNA was extracted from four biological replicates of 30 mixed sex individuals on each of the five *T. vaporariorum* cultures described above using the Isolate RNA mini-kit (Bioline) following the manufacturer’s protocol. RNA samples were checked for quality (A260/280 > 2.00, A260/230 > 1.8, > 150 ng/μL) and used as a template for the generation of barcoded libraries (TrueSeq RNA library preparation, Illumina) which were then sequenced to high coverage (~ 30 M PE reads per replicate) on an Illumina HiSeq2500 flowcell (125 bp paired end reads) at the Earlham Institute (Norwich, UK). All sequence data has been deposited with the NCBI Short Read archive as BioProject PRJNA548670.

The quality of the reads obtained was assessed using FASTQC v0.11.5 [85], and adaptor sequences and low quality base calls removed using TrimGalore 0.4.5 [86]. Clean reads were aligned to the genome using HISAT2 v2.1.0 [87], and gene expression estimated using the htseq-count tool implemented in the HTSeq package [88]. EdgeR v3.9 [89] was used to identify significantly differentially-expressed genes using a corrected *p*-value threshold of *p* < 0.05 and a fold change > 2. Comparisons were made between lists of differentially expressed genes using Venny v2.1.0 [90]. The expression of specific gene families, such as cytochrome P450s, across different treatments was visualised using heatmaps generated in RStudio [91]. Sequences were mapped and assigned Gene Ontology (GO) terms using Blast2GO [76] with gene set enrichment analysis performed using the GSEA software package [92].

### Transgenic expression of candidate genes in *D. melanogaster*

The *T. vaporariorum* P450 genes *CYP6CM2*, *CYP6CM3* and *CYP6CM4* were synthesised (GeneArt) and cloned into the pUASTattB plasmid (GenBank: EF362409.1). Using the PhiC31 system, constructs were transformed into the germline of a *D. melanogaster* strain carrying an attP docking site on chromosome 2 (attP40) and the phiC31 integrase gene under the control of the vasa regulatory region on the X chromosome (y w M (eGFP, vas-int, dmRFP)ZH-2A; P [CaryP]attP40) [93]. The transgenic lines obtained were balanced and the integration of genes confirmed by PCR and sequencing using Phusion DNA polymerase (Thermo) as described previously [94] with the primers detailed in Additional file 23: Table S24. Virgin females of the Act5C-GAL4 strain were crossed with UAS-gene-of-interest males. Bioassays were used to assess the susceptibility of adult female flies to nicotine and imidacloprid. Several concentrations were overlaid onto 1.5% agar containing 1% sucrose in standard *Drosophila* vials and allowed to dry overnight at room temperature. Twenty adult flies (2 to 5 days post eclosion) were then added to each vial and mortality assessed after 72 h. Five replicates were carried out for each concentration. Control mortality was assessed using vials containing agar/sucrose minus insecticide. LC_50_ values and 95% fiducial limits were calculated as above.

### Quantitative PCR

Primers for QPCR were designed to amplify a fragment of around 100 bp using the Primer3 plugin in Geneious (Additional file 23: Table S24). 1.5 μg of RNA was used for reverse transcription using the Maxima H Minus First Strand cDNA Synthesis Kit from Thermo Scientific (Waltham, MA, USA), adding both random hexamer and oligo (dT) primers. Each PCR reaction consisted of 5 μl of cDNA (3.125 ng), 7.5 μl of SYBR® Green JumpStart™ Taq ReadyMix™ (Sigma Aldrich, St. Louis, MO, USA) and 0.5 μl of each forward and reverse primer (0.25 μM). PCRs were run on a BioRad Real-Time PCR System with cycling conditions of: 2 min at 95 °C followed by 40 cycles of 95 °C for 30 s, 57 °C for 20 s and 72 °C for 25 s. A final melt-curve step was included post-PCR (ramping from 72 °C to 95 °C by 1 °C every 5 s) to confirm the absence of any non-specific amplification. The efficiency of PCR for each primer pair was assessed using a serial dilution from 100 ng to 0.01 ng of cDNA. Each qRT-PCR experiment consisted of four independent biological replicates with two technical replicates. Data were analysed according to the ΔΔCT method [95], using the geometric mean of two previously published housekeeping genes (*para* and EF1a [96]) for normalisation according to the strategy described previously [97].

## Supplementary information

**Additional file 1: Figure S1.** Phylogenetic relationship of *Solanum lycopersicum* (tomato), *Nicotiana tabacum* (tobacco), *Phaseolus vulgaris* (French bean), *Cucurbita pepo* (pumpkin) and cucumber (*Cucumis sativus*).

**Additional file 2: Table S1.** Summary of short-read DNAseq data.

**Additional file 3: Table S2.** *T. vaporariorum* genome characteristics.

**Additional file 4: Figure S2.** Analysis of the *T. vaporariorum* genome assembly. (A) Distribution of 19-mers obtained from *T. vaporariorum* DNA sequencing reads. The x-axis and y-axis correspond to the frequency of 19-mers. (B) Summary of Benchmarking Universal Single-Copy Orthologs (BUSCO) analysis of the *T. vaporariorum* genome assembly using Arthropoda, Eukaryote and Insecta BUSCO gene sets. (C) Functional annotation of the *T. vaporariorum* predicted gene models using BLAST and InterPro analysis.

**Additional file 5: Table S3.** 10X library and Supernova assembly statistics.

**Additional file 6: Table S4.** Summary of CEGMA (Core Eukaryotic Genes Mapping Approach) assessment.

**Additional file 7: Table S5.** Summary of gene annotation of the *T. vaporariorum* genome assembly.

**Additional file 8: Table S6.** Summary statistics from CAFE (Computational Analysis of gene Family Evolution) analysis.

**Additional file 9: Table S7.** Gene ontology (GO) terms significantly enriched in gene families specific to *T. vaporariorum* and *B. tabaci. (DOCX 13 kb)*

**Additional file 10: Table S8.** Gene ontology (GO) terms significantly enriched in gene families identified as rapidly evolving in *T. vaporariorum*.

**Additional file 11: Tables S9-S13.** Identifier and sequences of curated *T. vaporariorum* genes belonging to detoxification enzyme families. **Table S9** shows the P450 genes, **Table S10** the carboxyl/cholinesterases, **Table S11** the glutathione S-transferases, **Table S12** the UDP- glucuronosyltransferases and **Table S13** ATP-binding cassette transporters.

**Additional file 12: Table S14.** Number of P450, GST, CCE, UGT and ABC genes in the genomes of five insect species.

**Additional file 13: Figure S3.** The glutathione-S-transferase (GST) gene family of *T. vaporariorum*. (A) Maximum likelihood tree of GST genes from *T. vaporariorum* (red) and *B. tabaci* (black). Branches are coloured according to clade and bootstrap values of 1000 replicates are given as decimals on branches. (B) Relative expression (log2fold) of the full length GSTs from 4 *T. vaporariorum* lines compared to the French bean-reared line.

**Additional file 14: Figure S4.** The carboxyl/cholinesterases (CCE) gene family of *T. vaporariorum*. A) Maximum likelihood tree of CCE genes from *T. vaporariorum* (red) and *B. tabaci* (black). Branches are coloured according to clade and bootstrap values of 1000 replicates are given as decimals on branches. (B) Relative expression (log2fold) of the full length CCEs from 4 *T. vaporariorum* lines compared to the French bean-reared line.

**Additional file 15: Figure S5.** The ATP-binding cassette transporter (ABC transporter) gene family of *T. vaporariorum*. (A) Maximum likelihood tree of ABC transporter genes from *T. vaporariorum* (red) and *B. tabaci* (black). Branches are coloured according to clade and bootstrap values of 1000 replicates are given as decimals on branches. (B) Relative expression (log2fold) of the full length ABCs from 4 *T. vaporariorum* lines compared to the French bean-reared line.

**Additional file 16: Figure S6.** The UDP glucuronosyltransferases (UGT) gene family of *T. vaporariorum*. (A) Maximum likelihood tree of UGT genes from *T. vaporariorum* (red) and *B. tabaci* (black). Bootstrap values of 1000 replicates are given as percentages on branches. (B) Relative expression (log2fold) of the full length UGTs from 4 *T. vaporariorum* lines compared to the French bean-reared line.

**Additional file 17: Table S15**: Log dose probit mortality data for 5 lines of *T. vaporariorum* reared on different host plants to various insecticides.

**Additional file 18: Tables S16-S21** Genes identified as differentially expressed between lines of *T. vaporariorum* reared on different host plants when compared to a line reared on French bean. Each comparison is shown in a separate tab. **Table S16** shows genes differentially expressed between the cucumber and bean-reared line. **Table S17** shows genes differentially expressed between the pumpkin and bean-reared line. **Table S18** shows genes differentially expressed between the tomato and bean-reared line. **Table S19** shows genes differentially expressed between the tobacco and bean-reared line. **Table S20** shows genes that are commonly differentially expressed in both nightshade (tobacco and tomato) reared comparisons. **Table S21** shows genes that are commonly differentially expressed in both cucurbit (cucumber and pumpkin) reared comparisons.

**Additional file 19: Figure S7.** Gene ontology analysis of genes differentially expressed in the tobacco and tomato-reared lines of *T. vaporariorum*. Bars are coloured according to core or non-core processes.

**Additional file 20: Figure S8.** Validation of RNAseq analysis by quantitative PCR. The fold change in expression of 9 genes of *T. vaporariorum* on various host plants compared to the bean-reared line as calculated by RNAseq and QPCR analysis. Error bars on QPCR data indicate 95% confidence limits.

**Additional file 21: Table S22.** Number of genes belonging to the P450, GST, CCE, ABC or UGT superfamilies over/under-expressed in *T. vaporariorum* lines reared on different host plants (relative to a control line reared on French bean).

**Additional file 22: Table S23.** Log-dose probit mortality data for three insecticides against transgenic *D. melanogaster* expressing CYP6CM2, CYP6CM3 or CYP6CM4.

**Additional file 23: Table S24.** Sequence of oligonucleotide primers used in this study.

## Data Availability

The *T. vaporariorum* whole genome shotgun project has been deposited at DDBJ/ENA/GenBank under the accession VJOP00000000. The RNAseq data generated in this study has been deposited in the Sequence Read Archive (SRA) under accession PRJNA548670. Names and sequences of the genes manually curated in this study are shown in Additional file 18: Table S20, S21, Additional file 21: Table S22, Additional file 22: Table S23 and Additional file 23: Tables S24. The authors declare that all other data supporting the findings of this study are available within the article and its supplementary information files.

## Abbreviations

ABC transporter: ATP-binding cassette transporter
ANOVA: Analysis of variance
BLAST: Basic Local Alignment Search Tool
BUSCO: Benchmarking Universal Single-Copy Orthologues
CCE: Carboxyl/Cholinesterase
CEGMA: Core Eukaryotic genes mapping approach
DE: Differentially expressed
GO: Gene ontology
GPCR: G protein-coupled receptor
GST: Glutathione S-transferase
LC50: Lethal concentration 50
MEAM: Middle East Asia Minor I-II
MED: Mediterranean
MY: Millions of years
NCBI: National Center for Biotechnology Information
NHR: Nuclear hormone receptor
P450: Cytochrome P450
QPCR: Quantitative PCR
RNAseq: RNA sequencing
RR-2: Rebers and Riddiford subgroup 2
TR: Tolerance ratio
UGT: UDP-glucuronosyltransferase
ZF-TF: Zinc-finger-transciption factor
