# Supplementary figures and images for: Host plant adaptation in the polyphagous whitefly, Trialeurodes vaporariorum, is associated with transcriptional plasticity and altered sensitivity to insecticides
Source: BMC Genomics. 2019 Dec 19;20:996. doi: 10.1186/s12864-019-6397-3 (PMC6923851; doi:10.1186/s12864-019-6397-3)

# Additional File 1: Figure S1

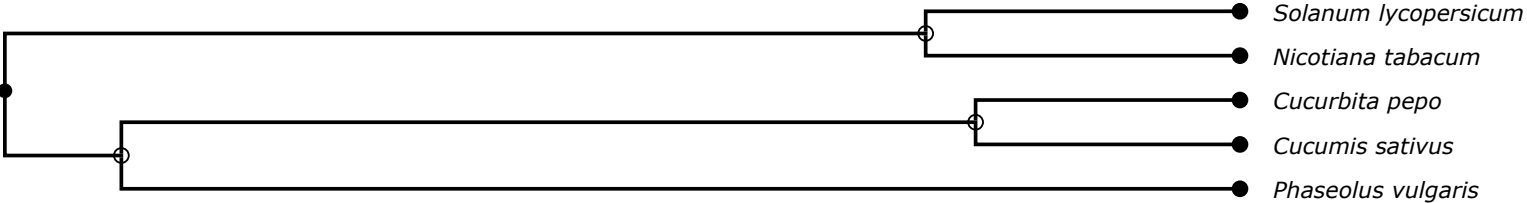

Supplement: Supplementary file 1 — Additional file 1: Figure S1. Phylogenetic relationship of Solanum lycopersicum (tomato), Nicotiana tabacum (tobacco), Phaseolus vulgaris (French bean), Cucurbita pepo (pumpkin) and cucumber (Cucumis sativus). [file 12864_2019_6397_MOESM1_ESM.pdf]

# Additional File: Figure S2

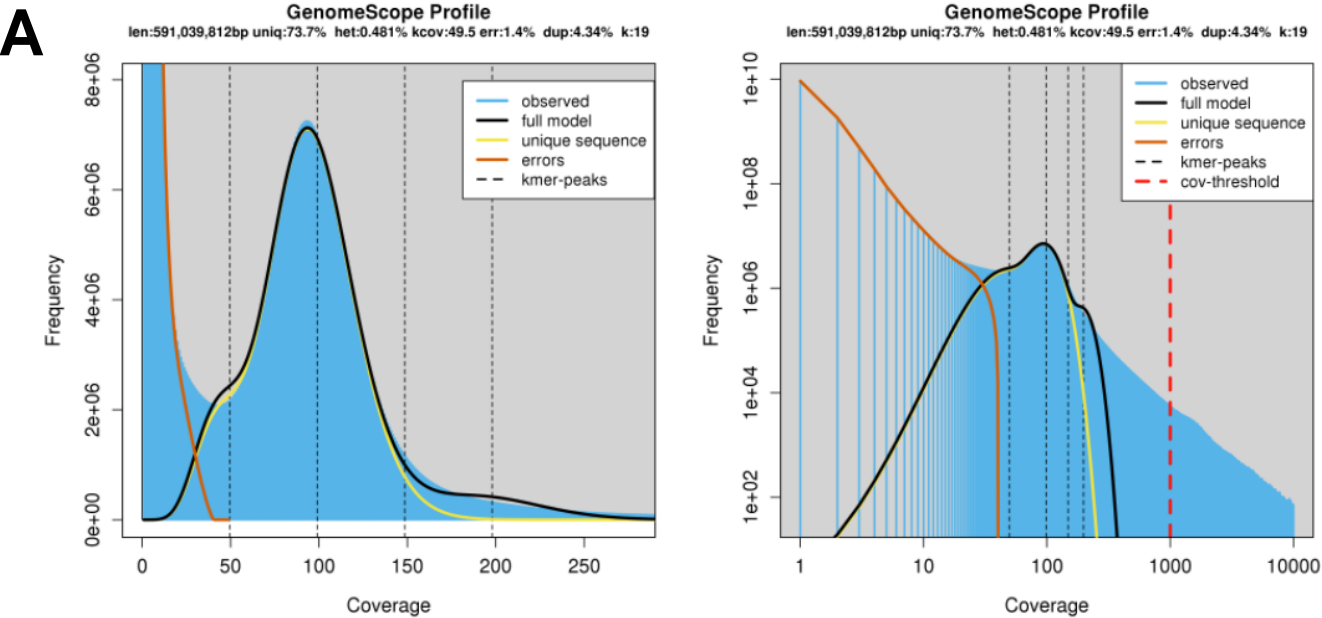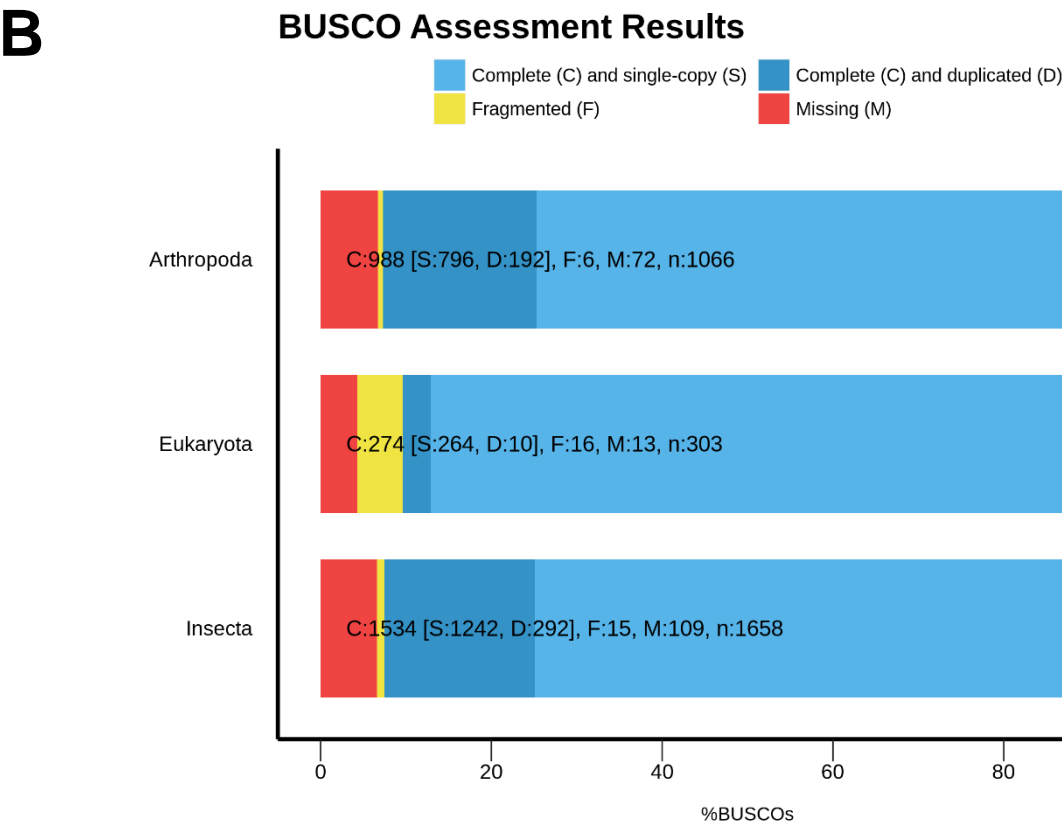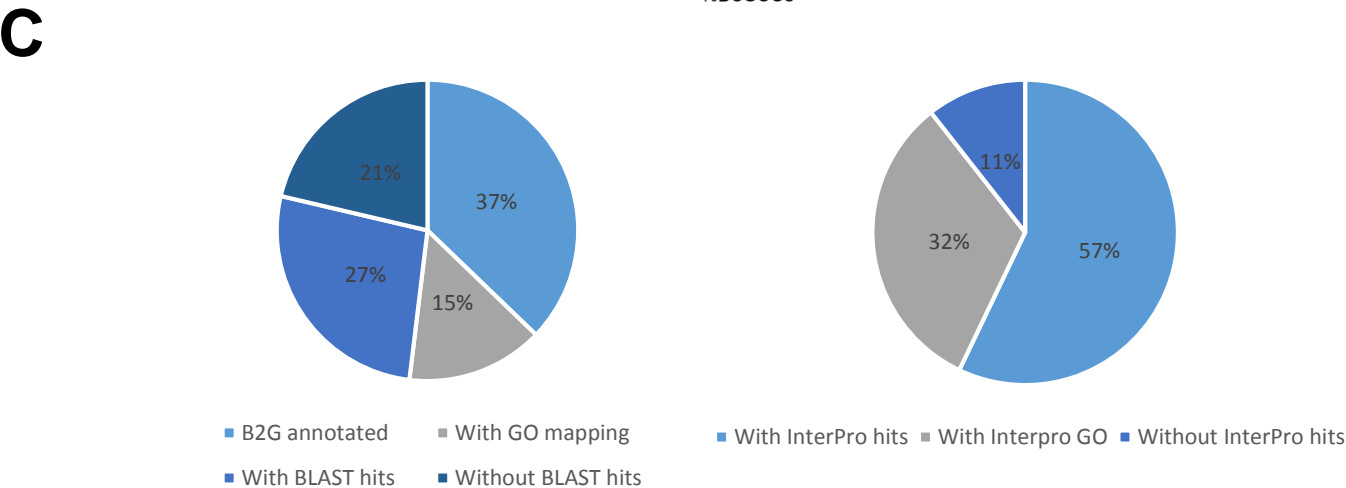

Supplement: Supplementary file 4 — Additional file 4: Figure S2. Analysis of the T. vaporariorum genome assembly. (A) Distribution of 19-mers obtained from T. vaporariorum DNA sequencing reads. The x-axis and y-axis correspond to the frequency of 19-mers. (B) Summary of Benchmarking Universal Single-Copy Orthologs (BUSCO) analysis of the T. vaporariorum genome assembly using Arthropoda, Eukaryote and Insecta BUSCO gene sets. (C) Functional annotation of the T. vaporariorum predicted gene models using BLAST and InterPro analysis. [file 12864_2019_6397_MOESM4_ESM.pdf]

Additional File 13: Figure S3

A

Delta

Microsomal

Sigma

Epsilon

Zeta

Omega

B

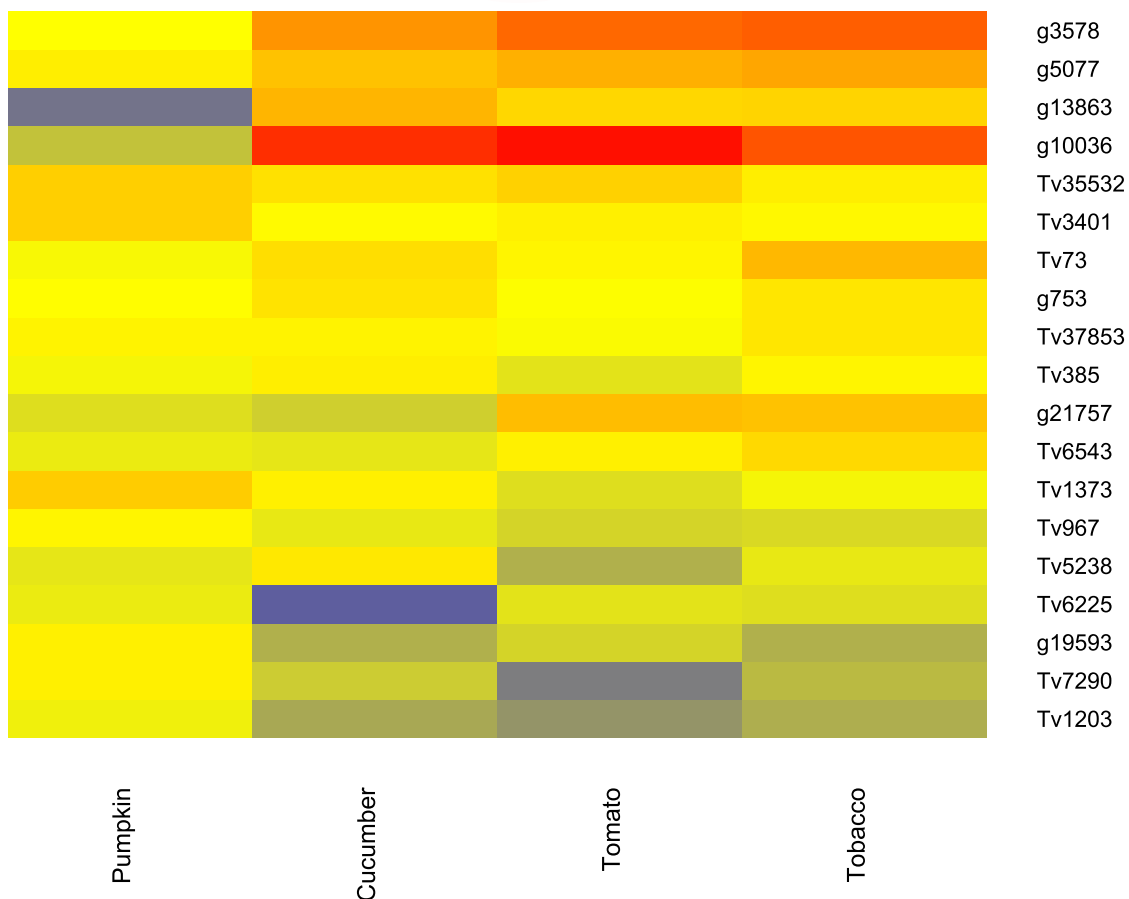

Supplement: Supplementary file 13 — Additional file 13: Figure S3. The glutathione-S-transferase (GST) gene family of T. vaporariorum. (A) Maximum likelihood tree of GST genes from T. vaporariorum (red) and B. tabaci (black). Branches are coloured according to clade and bootstrap values of 1000 replicates are given as decimals on branches. (B) Relative expression (log2fold) of the full length GSTs from 4 T. vaporariorum lines compared to the French bean-reared line. [file 12864_2019_6397_MOESM13_ESM.pdf]

Additional File 14: Figure S4

A

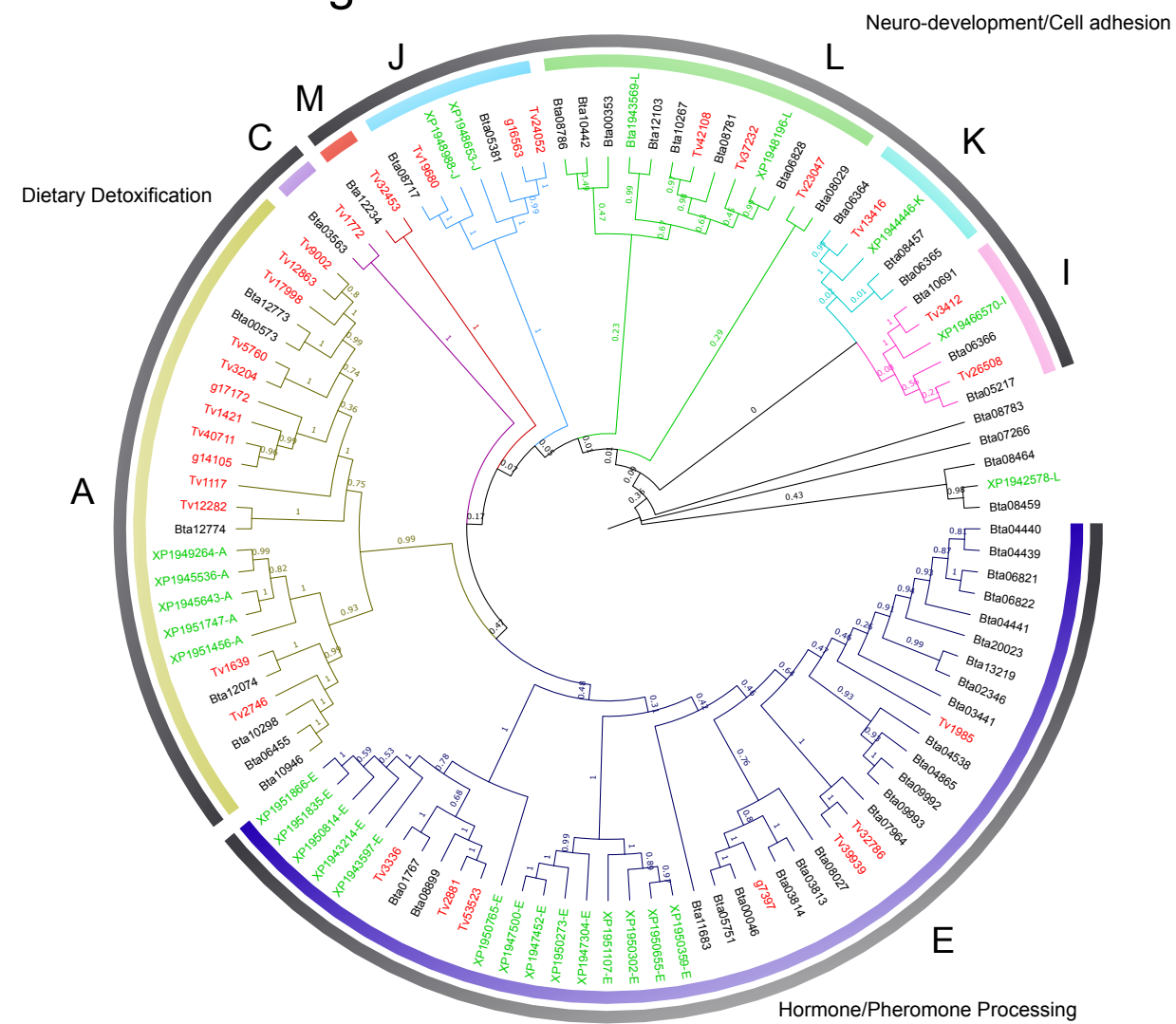

B

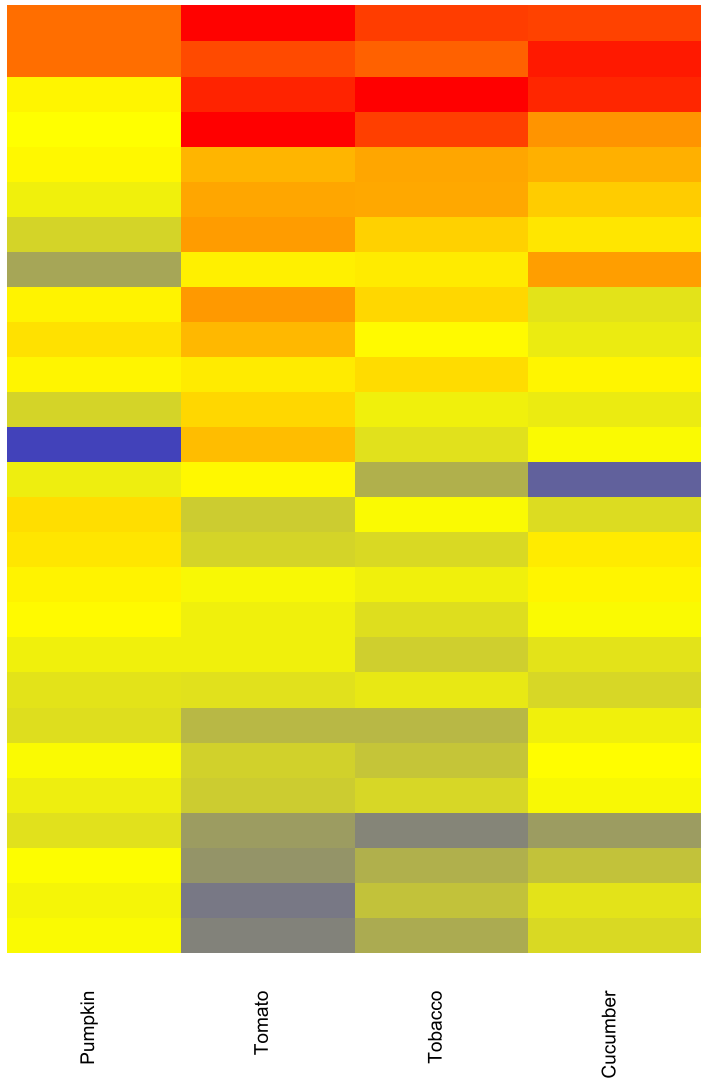

- Tv12863
- g14105
- g17172
- Tv37232
- Tv2881
- Tv42108
- Tv26508
- Tv40711
- Tv23047
- Tv12282
- Tv32453
- Tv9002
- Tv24052
- Tv19680
- g7397
- Tv3336
- Tv13416
- Tv1985
- Tv3412
- Tv1639
- Tv1117
- Tv1421
- g16563
- Tv1772
- Tv17998
- Tv53523
- Tv2746

Supplement: Supplementary file 14 — Additional file 14: Figure S4. The carboxyl/cholinesterases (CCE) gene family of T. vaporariorum. A) Maximum likelihood tree of CCE genes from T. vaporariorum (red) and B. tabaci (black). Branches are coloured according to clade and bootstrap values of 1000 replicates are given as decimals on branches. (B) Relative expression (log2fold) of the full length CCEs from 4 T. vaporariorum lines compared to the French bean-reared line. [file 12864_2019_6397_MOESM14_ESM.pdf]

Additional File 15: Figure S5

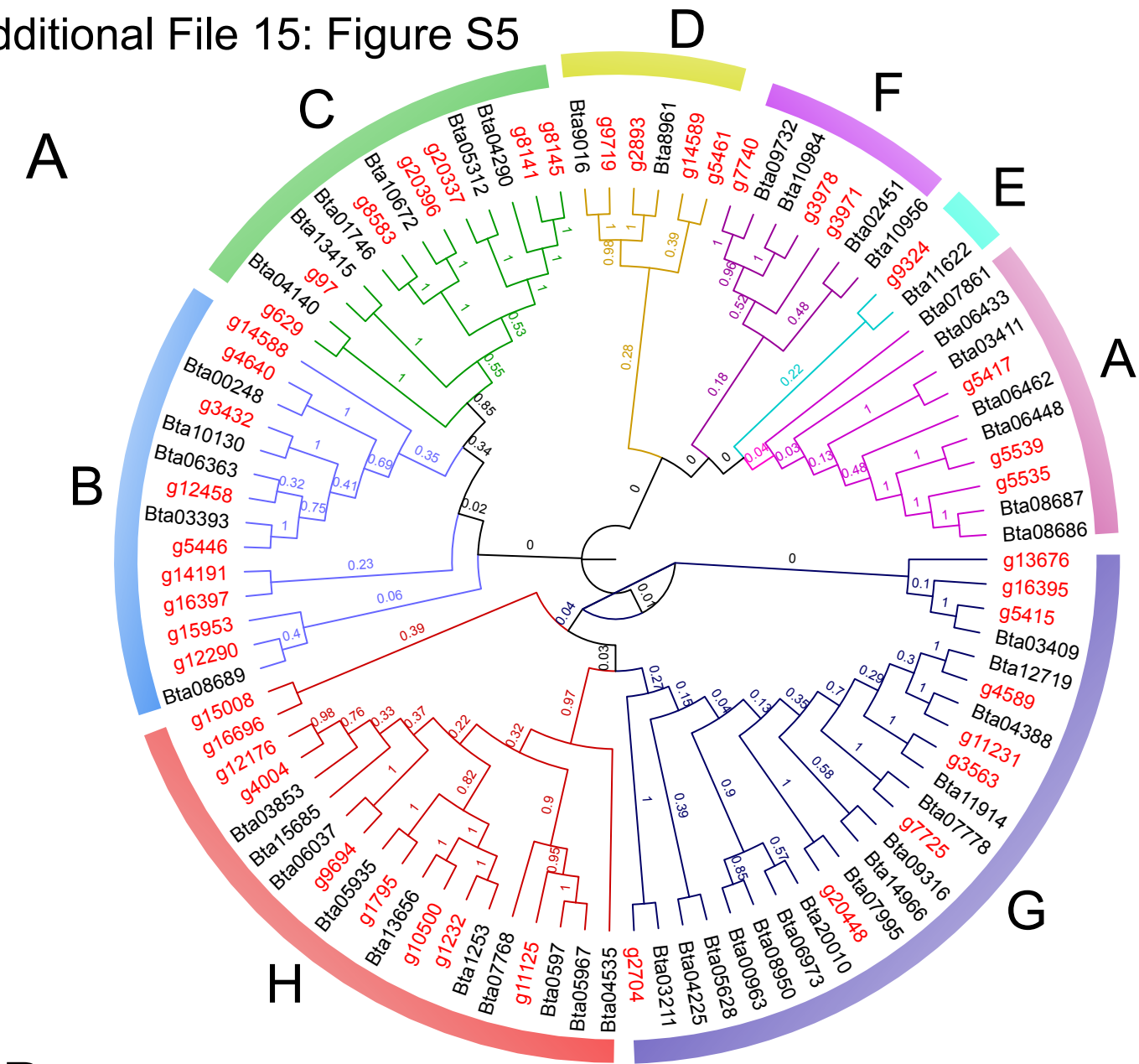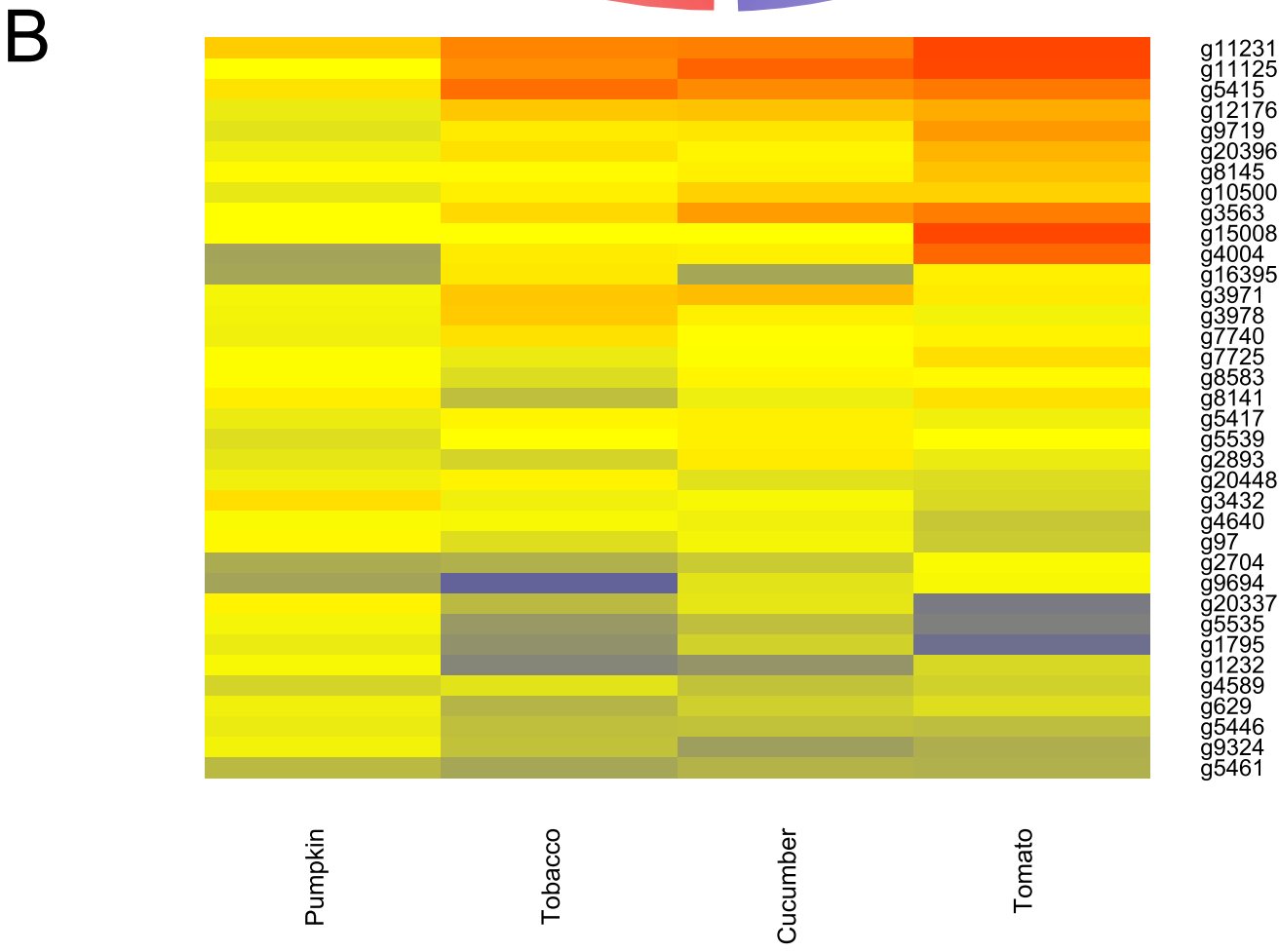

Supplement: Supplementary file 15 — Additional file 15: Figure S5. The ATP-binding cassette transporter (ABC transporter) gene family of T. vaporariorum. (A) Maximum likelihood tree of ABC transporter genes from T. vaporariorum (red) and B. tabaci (black). Branches are coloured according to clade and bootstrap values of 1000 replicates are given as decimals on branches. (B) Relative expression (log2fold) of the full length ABCs from 4 T. vaporariorum lines compared to the French bean-reared line. [file 12864_2019_6397_MOESM15_ESM.pdf]

# Additional File 16: Figure S6

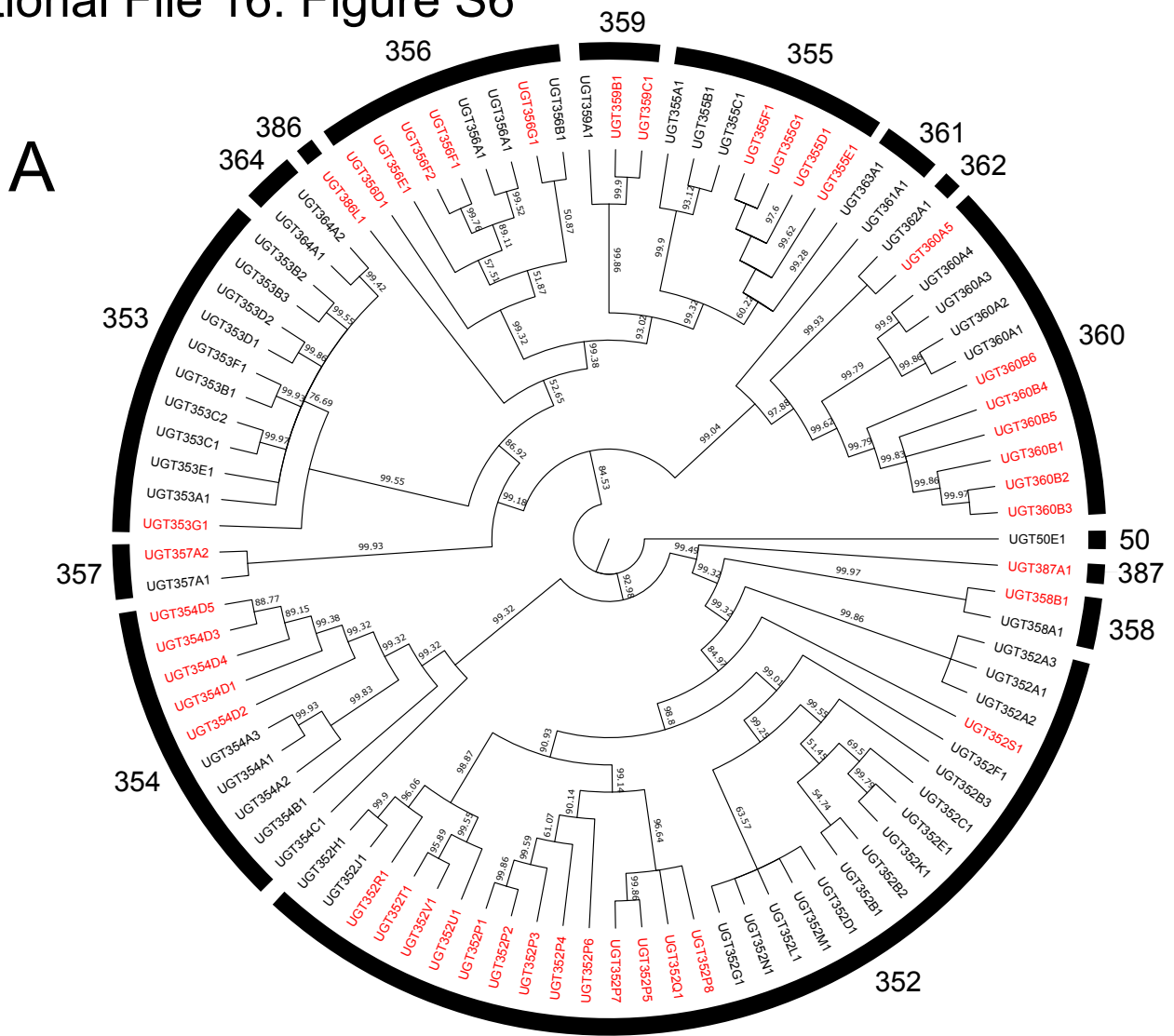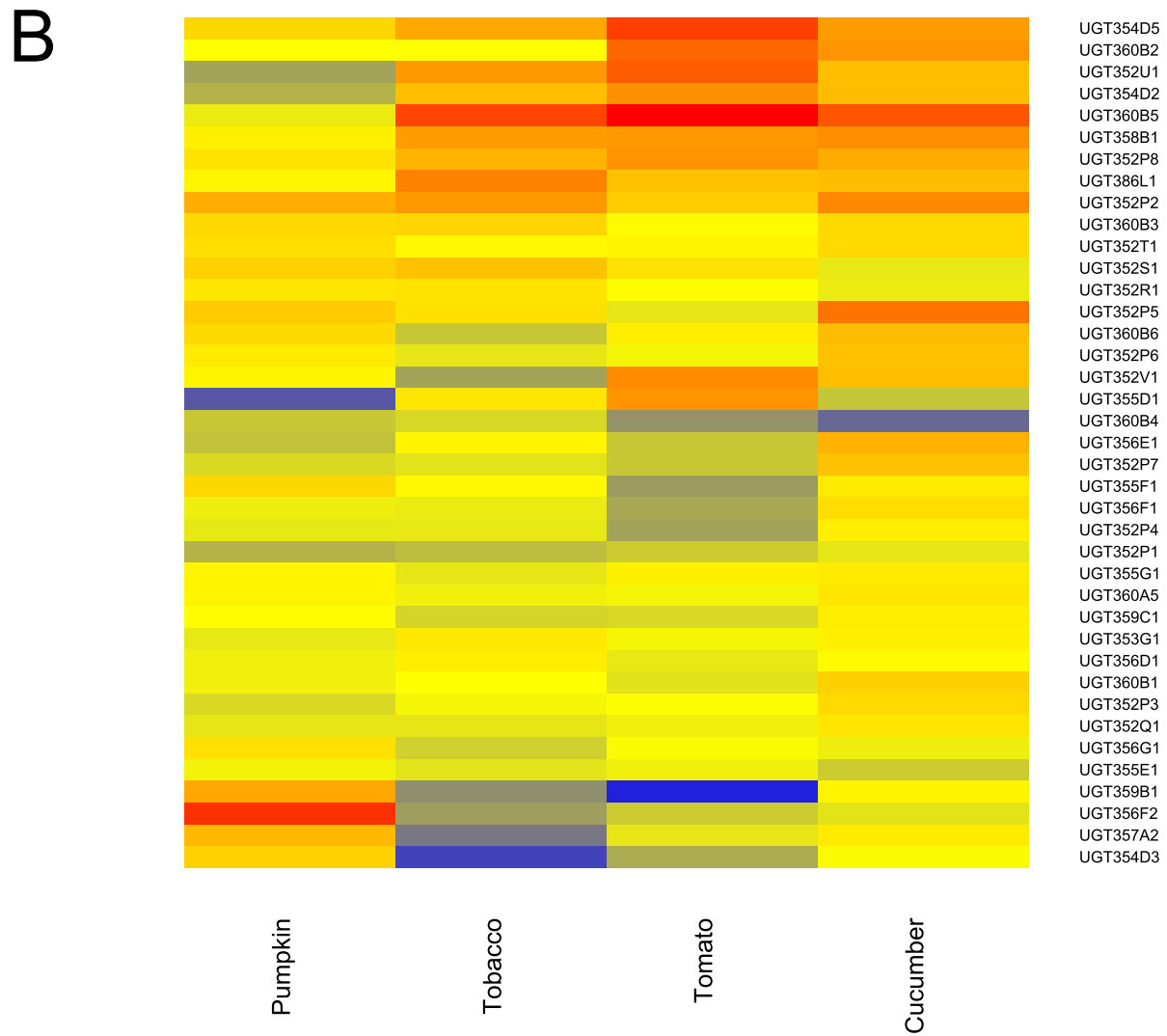

Supplement: Supplementary file 16 — Additional file 16: Figure S6. The UDP glucuronosyltransferases (UGT) gene family of T. vaporariorum. (A) Maximum likelihood tree of UGT genes from T. vaporariorum (red) and B. tabaci (black). Bootstrap values of 1000 replicates are given as percentages on branches. (B) Relative expression (log2fold) of the full length UGTs from 4 T. vaporariorum lines compared to the French bean-reared line. [file 12864_2019_6397_MOESM16_ESM.pdf]

# Additional File 19: Figure S7

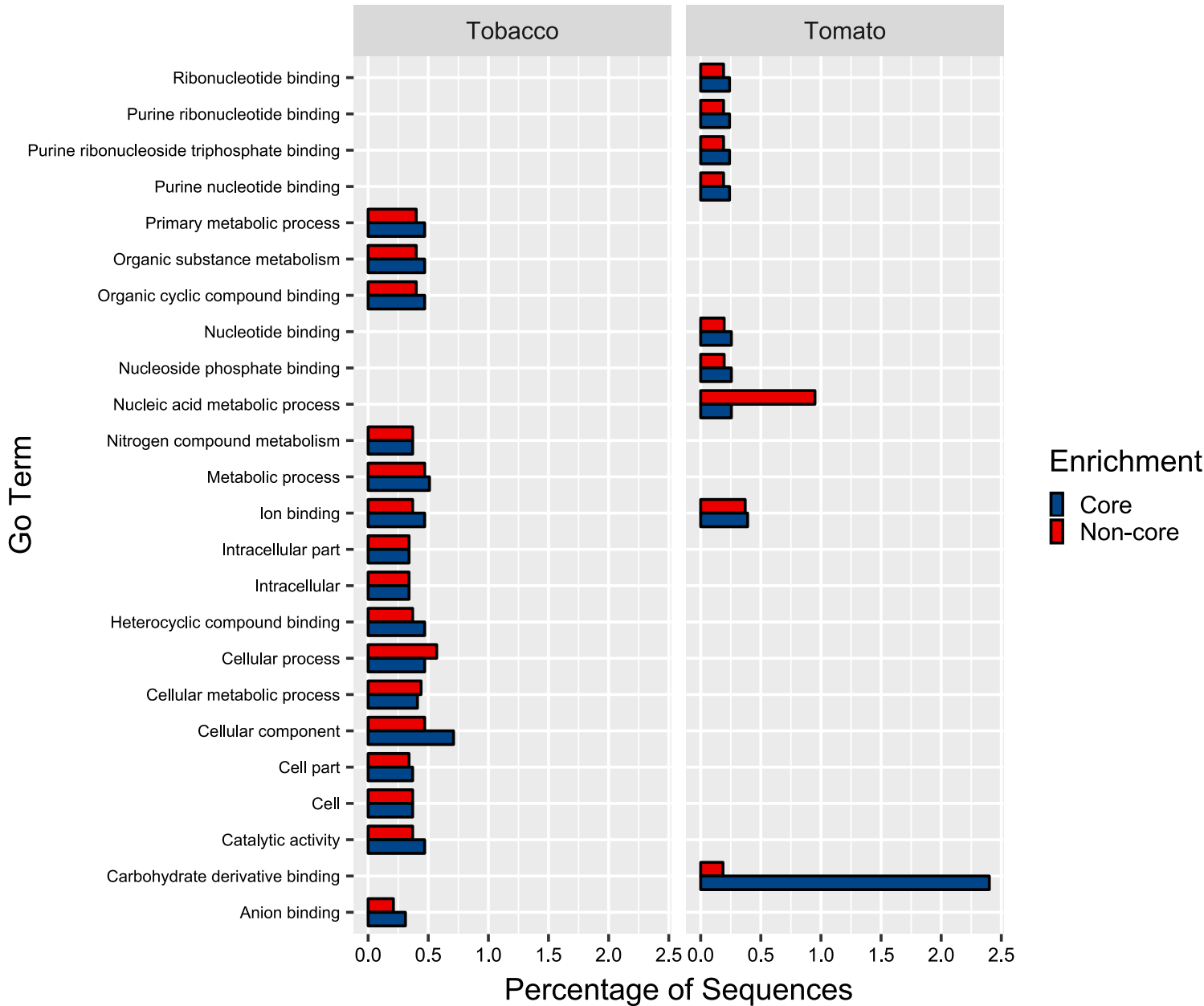

Supplement: Supplementary file 19 — Additional file 19: Figure S7. Gene ontology analysis of genes differentially expressed in the tobacco and tomato-reared lines of T. vaporariorum. Bars are coloured according to core or non-core processes. [file 12864_2019_6397_MOESM19_ESM.pdf]

Additional File 20: Figure S8

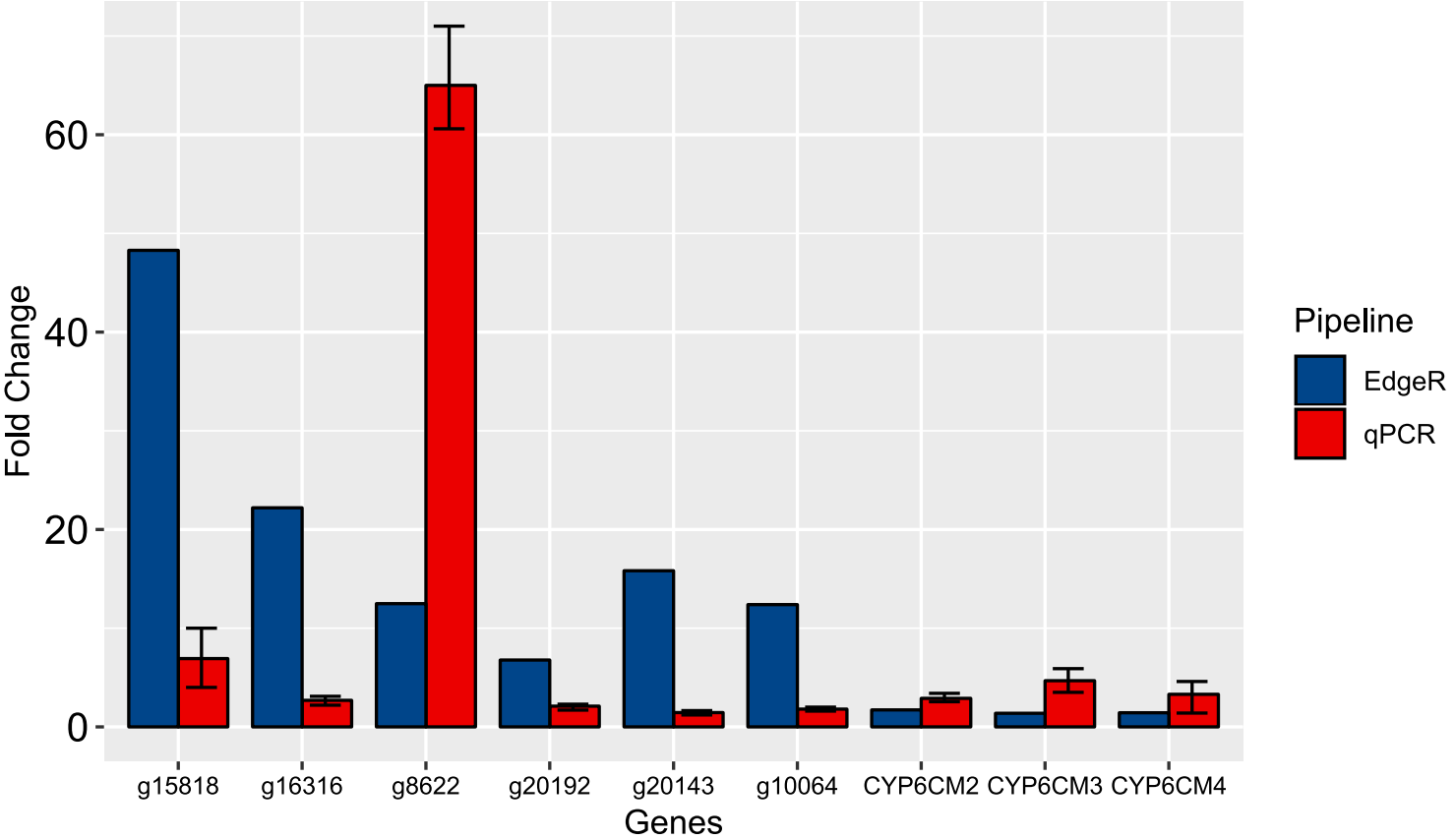

Supplement: Supplementary file 20 — Additional file 20: Figure S8. Validation of RNAseq analysis by quantitative PCR. The fold change in expression of 9 genes of T. vaporariorum on various host plants compared to the bean-reared line as calculated by RNAseq and QPCR analysis. Error bars on QPCR data indicate 95% confidence limits. [file 12864_2019_6397_MOESM20_ESM.pdf]
