# Supplementary material for: Host plant adaptation in the polyphagous whitefly, Trialeurodes vaporariorum, is associated with transcriptional plasticity and altered sensitivity to insecticides
Source: BMC Genomics. 2019 Dec 19;20:996. doi: 10.1186/s12864-019-6397-3 (PMC6923851; doi:10.1186/s12864-019-6397-3)
Supplement: Supplementary file 2 — Additional file 2: Table S1. Summary of short-read DNAseq data. [file 12864_2019_6397_MOESM2_ESM.docx]

**Additional File 2: Table S1**: Summary of short-read DNAseq data

| **Library** | **Type** | **Raw reads** | **Raw bases** | **Insert size** | **GC%** |
| --- | --- | --- | --- | --- | --- |
| Tv1_S1_L001 | PE | 396751198 x 2 | 119818861796 | 250 | 41 |
| Tv1_S1_L002 | PE | 394903809 x 2 | 119260950318 | 250 | 41 |
