# Supplementary material for: Host plant adaptation in the polyphagous whitefly, Trialeurodes vaporariorum, is associated with transcriptional plasticity and altered sensitivity to insecticides
Source: BMC Genomics. 2019 Dec 19;20:996. doi: 10.1186/s12864-019-6397-3 (PMC6923851; doi:10.1186/s12864-019-6397-3)
Supplement: Supplementary file 3 — Additional file 3: Table S2. T. vaporariorum genome characteristics. [file 12864_2019_6397_MOESM3_ESM.docx]

**Additional File 3: Table S2**: *T. vaporariorum* genome characteristics

| **Property** | **Minimum** | **Maximum** |
| --- | --- | --- |
| Heterozygosity | 0.47% | 0.49% |
| Genome Haploid Length | 589,111,869 bp | 591,039,812 bp |
| Genome Repeat Length | 154,680,419 bp | 155,186,630 bp |
| Genome Unique Length | 434,431,450 bp | 435,853,182 bp |
| Model Fit | 92.7527% | 96.1024% |
| Read Error Rate | 1.39693% | 1.39693% |
