# Supplementary material for: Host plant adaptation in the polyphagous whitefly, Trialeurodes vaporariorum, is associated with transcriptional plasticity and altered sensitivity to insecticides
Source: BMC Genomics. 2019 Dec 19;20:996. doi: 10.1186/s12864-019-6397-3 (PMC6923851; doi:10.1186/s12864-019-6397-3)
Supplement: Supplementary file 5 — Additional file 5: Table S3. 10X library and Supernova assembly statistics. [file 12864_2019_6397_MOESM5_ESM.docx]

**Additional File 5: Table S3**: 10X library and Supernova assembly statistics

| **Property** | **Value** |
| --- | --- |
| Number of reads | 300.02 M |
| Mean read length after trimming | 139.50 |
| Raw coverage | 51.32x |
| Effective read coverage | 40.92x |
| Fraction of Q30 bases in read 2 | 83.44% |
| Median insert size | 367 bp |
| Fraction of proper read pairs | 87.92% |
| Fraction of barcodes used | 0.50 |
| Estimated repetitive fraction | 14.04% |
| Weighted mean molecule size | 33.75 Kb |
| Molecule count extending 10 kb on both sides | 99.18 |
| Mean distance between heterozygous SNPs | 477.00 b |
| Fraction of reads that are not barcoded | 4.27% |
| Nonduplicate and phased reads | 57.25% |
| Number of scaffolds >= 10 kb | 6.02 K |
| N50 edge size | 4.05 Kb |
| N50 contig size | 21.67 Kb |
| N50 phase block size | 333.00 Kb |
| N50 scaffold size | 921.58 Kb |
| Assembly size (only scaffolds >= 10 kb) | 581.92 Mb |
| % of base assembly missing from scaffolds >= 10 kb | 23.15% |
| High AT index | 0.17% |
