# Supplementary material for: Host plant adaptation in the polyphagous whitefly, Trialeurodes vaporariorum, is associated with transcriptional plasticity and altered sensitivity to insecticides
Source: BMC Genomics. 2019 Dec 19;20:996. doi: 10.1186/s12864-019-6397-3 (PMC6923851; doi:10.1186/s12864-019-6397-3)
Supplement: Supplementary file 6 — Additional file 6: Table S4. Summary of CEGMA (Core Eukaryotic Genes Mapping Approach) assessment. [file 12864_2019_6397_MOESM6_ESM.docx]

**Additional file 6: Table S4:** Summary of CEGMA (Core Eukaryotic Genes Mapping Approach) assessment

| **Category** | **#Prots** | **%completeness** | **#Total** | **Avg.** | **#Ortho** |
| --- | --- | --- | --- | --- | --- |
| Complete | 173 | 69.76 | 209 | 1.21 | 16.76 |
| Partial | 235 | 94.76 | 383 | 1.63 | 44.68 |
