# Supplementary material for: Host plant adaptation in the polyphagous whitefly, Trialeurodes vaporariorum, is associated with transcriptional plasticity and altered sensitivity to insecticides
Source: BMC Genomics. 2019 Dec 19;20:996. doi: 10.1186/s12864-019-6397-3 (PMC6923851; doi:10.1186/s12864-019-6397-3)
Supplement: Supplementary file 7 — Additional file 7: Table S5. Summary of gene annotation of the T. vaporariorum genome assembly. [file 12864_2019_6397_MOESM7_ESM.docx]

**Additional file 7: Table S5**: Summary of gene annotation of the *T. vaporariorum* genome assembly

| **Property** | **Value** |
| --- | --- |
| Total sequence length | 896220028 |
| Number of genes | 22735 |
| Number of mRNAs | 24327 |
| Number of exons | 122773 |
| Number of introns | 98446 |
| Number of CDS | 24327 |
| Overlapping genes | 230 |
| Contained genes | 87 |
| CDS: no stop, no start | 24327 |
| Total gene length | 249417867 |
| Total mRNA length | 309075704 |
| Total exon length | 30768638 |
| Total intron length | 272260430 |
| Total CDS length | 30768638 |
| Shortest gene | 55 |
| Shortest mRNA | 55 |
| Shortest exon | 3 |
| Shortest intron | 42 |
| Shortest CDS | 23 |
| Longest gene | 364052 |
| Longest mRNA | 364052 |
| Longest exon | 13746 |
| Longest intron | 146505 |
| Longest CDS | 38118 |
| mean gene length | 10971 |
| mean mRNA length | 12705 |
| mean exon length | 251 |
| mean intron length | 2766 |
| mean CDS length | 1265 |
| % of genome covered by genes | 27.8 |
| % of genome covered by CDS | 3.4 |
| mean mRNAs per gene | 1 |
| mean exons per mRNA | 5 |
| mean introns per mRNA | 4 |
