# Supplementary material for: Host plant adaptation in the polyphagous whitefly, Trialeurodes vaporariorum, is associated with transcriptional plasticity and altered sensitivity to insecticides
Source: BMC Genomics. 2019 Dec 19;20:996. doi: 10.1186/s12864-019-6397-3 (PMC6923851; doi:10.1186/s12864-019-6397-3)
Supplement: Supplementary file 8 — Additional file 8: Table S6. Summary statistics from CAFE (Computational Analysis of gene Family Evolution) analysis. [file 12864_2019_6397_MOESM8_ESM.docx]

**Additional file 8: Table S6**: Summary statistics from CAFE (Computational Analysis of gene Family Evolution) analysis

| **Species** | **Expanded families** | **Genes gained** | **Genes/ expansion** | **Contracted families** | **Genes lost** | **Genes/ contracted** | **No change** | **Average expansion** |
| --- | --- | --- | --- | --- | --- | --- | --- | --- |
| *T.vaporariorum* | 1832 (40 | 2931 | 1.6 | 587(3) | 734 | 1.25 | 12978 | 0.1427 |
| *D.melanogaster* | 4234 (21) | 9845 | 2.33 | 8295(10) | 9117 | 1.1 | 2868 | 0.0473 |
| *M.persicae* | 4553 (89) | 7056 | 1.55 | 1476(131) | 2619 | 1.77 | 9368 | 0.2882 |
| *B.tabaci* | 545 (15) | 1070 | 1.96 | 2213(21) | 2600 | 1,17 | 12639 | -0.0993 |
| *A.nogla* | 970 (94) | 3517 | 3.63 | 2152(7) | 2921 | 1.36 | 12275 | 0.0387 |
| *T.castaneum* | 2888 (32) | 6312 | 2.19 | 1415(28) | 2061 | 1.46 | 11094 | 0.2761 |
| *A.pisum* | 1482 (200) | 4919 | 3.32 | 2968 (64) | 3573 | 1.2 | 10947 | 0.0874 |
