# Supplementary material for: Host plant adaptation in the polyphagous whitefly, Trialeurodes vaporariorum, is associated with transcriptional plasticity and altered sensitivity to insecticides
Source: BMC Genomics. 2019 Dec 19;20:996. doi: 10.1186/s12864-019-6397-3 (PMC6923851; doi:10.1186/s12864-019-6397-3)
Supplement: Supplementary file 9 — Additional file 9: Table S7. Gene ontology (GO) terms significantly enriched in gene families specific to T. vaporariorum and B. tabaci. (DOCX 13 kb) [file 12864_2019_6397_MOESM9_ESM.docx]

**Additional file 9: Table S7**: Gene ontology (GO) terms significantly enriched in gene families specific to *T. vaporariorum* and *B. tabaci*

| Tags | GO ID | GO Name | GO Category | FDR |
| --- | --- | --- | --- | --- |
| [OVER] | GO:0005975 | carbohydrate metabolic process | BIOLOGICAL_PROCESS | 8.26E-17 |
| [OVER] | GO:0008234 | cysteine-type peptidase activity | MOLECULAR_FUNCTION | 9.87E-13 |
| [OVER] | GO:0070011 | peptidase activity, acting on L-amino acid peptides | MOLECULAR_FUNCTION | 2.93E-04 |
| [OVER] | GO:0008233 | peptidase activity | MOLECULAR_FUNCTION | 3.98E-04 |
| [OVER] | GO:0006508 | proteolysis | BIOLOGICAL_PROCESS | 6.31E-04 |
| [OVER] | GO:0004869 | cysteine-type endopeptidase inhibitor activity | MOLECULAR_FUNCTION | 0.00157 |
| [OVER] | GO:0008270 | zinc ion binding | MOLECULAR_FUNCTION | 0.008193 |
| [OVER] | GO:0016758 | transferase activity, transferring hexosyl groups | MOLECULAR_FUNCTION | 0.028012 |
