# Supplementary material for: Host plant adaptation in the polyphagous whitefly, Trialeurodes vaporariorum, is associated with transcriptional plasticity and altered sensitivity to insecticides
Source: BMC Genomics. 2019 Dec 19;20:996. doi: 10.1186/s12864-019-6397-3 (PMC6923851; doi:10.1186/s12864-019-6397-3)
Supplement: Supplementary file 10 — Additional file 10: Table S8. Gene ontology (GO) terms significantly enriched in gene families identified as rapidly evolving in T. vaporariorum. [file 12864_2019_6397_MOESM10_ESM.docx]

**Additional file 10: Table S8**: Gene ontology (GO) terms significantly enriched in gene families identified as rapidly evolving in *T. vaporariorum*

| Tags | GO ID | GO Name | GO Category | FDR |
| --- | --- | --- | --- | --- |
| [OVER] | GO:0006259 | DNA metabolic process | BIOLOGICAL_PROCESS | 2.73E-20 |
| [OVER] | GO:0090304 | nucleic acid metabolic process | BIOLOGICAL_PROCESS | 4.15E-16 |
| [OVER] | GO:0006139 | nucleobase-containing compound metabolic process | BIOLOGICAL_PROCESS | 1.79E-14 |
| [OVER] | GO:0046483 | heterocycle metabolic process | BIOLOGICAL_PROCESS | 4.62E-14 |
| [OVER] | GO:0044260 | cellular macromolecule metabolic process | BIOLOGICAL_PROCESS | 4.62E-14 |
| [OVER] | GO:0006725 | cellular aromatic compound metabolic process | BIOLOGICAL_PROCESS | 5.61E-14 |
| [OVER] | GO:1901360 | organic cyclic compound metabolic process | BIOLOGICAL_PROCESS | 6.37E-14 |
| [OVER] | GO:0043170 | macromolecule metabolic process | BIOLOGICAL_PROCESS | 7.76E-14 |
| [OVER] | GO:0034641 | cellular nitrogen compound metabolic process | BIOLOGICAL_PROCESS | 9.07E-13 |
| [OVER] | GO:0006807 | nitrogen compound metabolic process | BIOLOGICAL_PROCESS | 5.63E-12 |
| [OVER] | GO:0044238 | primary metabolic process | BIOLOGICAL_PROCESS | 1.49E-11 |
| [OVER] | GO:0015074 | DNA integration | BIOLOGICAL_PROCESS | 1.49E-11 |
| [OVER] | GO:0044237 | cellular metabolic process | BIOLOGICAL_PROCESS | 9.23E-11 |
| [OVER] | GO:0071704 | organic substance metabolic process | BIOLOGICAL_PROCESS | 1.06E-10 |
| [OVER] | GO:0003676 | nucleic acid binding | MOLECULAR_FUNCTION | 1.26E-10 |
| [OVER] | GO:0006313 | transposition, DNA-mediated | BIOLOGICAL_PROCESS | 1.59E-10 |
| [OVER] | GO:0032196 | transposition | BIOLOGICAL_PROCESS | 1.59E-10 |
| [OVER] | GO:0006310 | DNA recombination | BIOLOGICAL_PROCESS | 1.76E-09 |
| [OVER] | GO:0009987 | cellular process | BIOLOGICAL_PROCESS | 5.40E-09 |
| [OVER] | GO:0008152 | metabolic process | BIOLOGICAL_PROCESS | 5.40E-09 |
| [OVER] | GO:0004803 | transposase activity | MOLECULAR_FUNCTION | 1.34E-07 |
| [OVER] | GO:1901363 | heterocyclic compound binding | MOLECULAR_FUNCTION | 2.47E-07 |
| [OVER] | GO:0140097 | catalytic activity, acting on DNA | MOLECULAR_FUNCTION | 2.47E-07 |
| [OVER] | GO:0097159 | organic cyclic compound binding | MOLECULAR_FUNCTION | 2.75E-07 |
| [OVER] | GO:0005488 | binding | MOLECULAR_FUNCTION | 6.19E-06 |
| [OVER] | GO:0000943 | retrotransposon nucleocapsid | CELLULAR_COMPONENT | 4.92E-05 |
| [OVER] | GO:0044428 | nuclear part | CELLULAR_COMPONENT | 1.24E-04 |
| [OVER] | GO:0008270 | zinc ion binding | MOLECULAR_FUNCTION | 1.77E-04 |
| [OVER] | GO:0046914 | transition metal ion binding | MOLECULAR_FUNCTION | 0.002396 |
| [OVER] | GO:0044446 | intracellular organelle part | CELLULAR_COMPONENT | 0.015702 |
| [OVER] | GO:0005634 | nucleus | CELLULAR_COMPONENT | 0.017706 |
| [OVER] | GO:0044422 | organelle part | CELLULAR_COMPONENT | 0.021433 |
| [OVER] | GO:0003824 | catalytic activity | MOLECULAR_FUNCTION | 0.033101 |
| [OVER] | GO:0043231 | intracellular membrane-bounded organelle | CELLULAR_COMPONENT | 0.035243 |
| [OVER] | GO:0043227 | membrane-bounded organelle | CELLULAR_COMPONENT | 0.039062 |
