# Supplementary material for: Host plant adaptation in the polyphagous whitefly, Trialeurodes vaporariorum, is associated with transcriptional plasticity and altered sensitivity to insecticides
Source: BMC Genomics. 2019 Dec 19;20:996. doi: 10.1186/s12864-019-6397-3 (PMC6923851; doi:10.1186/s12864-019-6397-3)
Supplement: Supplementary file 12 — Additional file 12: Table S14. Number of P450, GST, CCE, UGT and ABC genes in the genomes of five insect species. [file 12864_2019_6397_MOESM12_ESM.docx]

**Additional file 12: Table S14**: Number of P450, GST, CCE, UGT and ABC genes in the genomes of five insect species.

| **Species** | **P450s** | **GSTs** | **CCEs** | **UGTs** | **ABCs** | **Total** |
| --- | --- | --- | --- | --- | --- | --- |
| *T. castaneum* | 128 | 35 | 60 | 27 | 73 | 323 |
| 1. *pisum* | 85 | 23 | 49 | 71 | 126 | 354 |
| 1. *tabaci* | 130 | 24 | 51 | 51 | 50 | 306 |
| *D. melanogaster* | 91 | 39 | 35 | 33 | 56 | 254 |
| *T. vaporariorum* | 80(+23) | 26(+4) | 31(+4) | 42 | 46 | 225 |
