# Supplementary material for: Host plant adaptation in the polyphagous whitefly, Trialeurodes vaporariorum, is associated with transcriptional plasticity and altered sensitivity to insecticides
Source: BMC Genomics. 2019 Dec 19;20:996. doi: 10.1186/s12864-019-6397-3 (PMC6923851; doi:10.1186/s12864-019-6397-3)
Supplement: Supplementary file 17 — Additional file 17: Table S15: Log dose probit mortality data for 5 lines of T. vaporariorum reared on different host plants to various insecticides. [file 12864_2019_6397_MOESM17_ESM.docx]

**Additional file 17: Table S15**: Log dose probit mortality data for 5 lines of *T. vaporariorum* reared on different host plants to various insecticides

| **Chemical** | **Line** | **LC_50_ Value (95% CI) (mgL^-1^)** |
| --- | --- | --- |
| Nicotine | Bean | 797.99 (528.03-1191.82) |
|  | Pumpkin | 3430.14 (1814-6485.86) |
|  | Cucumber | 707.71 (538.03-1191.82) |
|  | Tobacco | 3981 (2072-7648.14) |
|  | Tomato | 1072 (772-1491) |
| Imidacloprid | Bean | 0.39 (0.21-0.52) |
|  | Pumpkin | 0.48 (0.34-0.69) |
|  | Cucumber | 0.43 (0.32-0.58) |
|  | Tobacco | 0.73 (0.51-1.05) |
|  | Tomato | 1.25 (0.97-1.6) |
| Chlorantraniliprole | Bean | 1.57 (0.12-3.68) |
|  | Pumpkin | 20.34 (10.37-61.18) |
|  | Cucumber | 66.65 (29.78-507.55) |
|  | Tobacco | 5.41 (2.47-9.42) |
|  | Tomato | 1.21 (0.49-3.3) |
| Pymetrozine | Bean | 119.7 (17.6-816) |
|  | Pumpkin | 5.1 (0.9-29.8) |
|  | Cucumber | 134.8 (23.8-788.9) |
|  | Tobacco | 780.7 (351-2676.3) |
|  | Tomato | 321.66 (242.66-427.66) |
| Bifenthrin | Bean | 0.4 (0.04-1.8) |
|  | Pumpkin | 3.4 (1.8-6.7) |
|  | Cucumber | 3.2 (1.1-13.4) |
|  | Tobacco | 6.9 (3.5-13.9) |
|  | Tomato | 6.38 (2-20.12) |
