# Supplementary material for: Host plant adaptation in the polyphagous whitefly, Trialeurodes vaporariorum, is associated with transcriptional plasticity and altered sensitivity to insecticides
Source: BMC Genomics. 2019 Dec 19;20:996. doi: 10.1186/s12864-019-6397-3 (PMC6923851; doi:10.1186/s12864-019-6397-3)
Supplement: Supplementary file 21 — Additional file 21: Table S22. Number of genes belonging to the P450, GST, CCE, ABC or UGT superfamilies over/under-expressed in T. vaporariorum lines reared on different host plants (relative to a control line reared on French bean). [file 12864_2019_6397_MOESM21_ESM.docx]

**Additional file 21: Table S22**: Number of genes belonging to the P450, GST, CCE, ABC or UGT superfamilies over/under-expressed in *T. vaporariorum* lines reared on different host plants (relative to a control line reared on French bean)

| **Line** | **P450** | **GST** | **CCE** | **ABC** | **UGT** |
| --- | --- | --- | --- | --- | --- |
| Cucumber | 8/3 | 1/1 | 2/2 | 4/2 | 5/0 |
| Pumpkin | 0/0 | 0/0 | 0/0 | 0/0 | 1/0 |
| Tobacco | 8/11 | 2/1 | 3/3 | 2/4 | 4/1 |
| Tomato | 16/12 | 2/0 | 5/5 | 5/5 | 5/5 |
