# Supplementary material for: Host plant adaptation in the polyphagous whitefly, Trialeurodes vaporariorum, is associated with transcriptional plasticity and altered sensitivity to insecticides
Source: BMC Genomics. 2019 Dec 19;20:996. doi: 10.1186/s12864-019-6397-3 (PMC6923851; doi:10.1186/s12864-019-6397-3)
Supplement: Supplementary file 22 — Additional file 22: Table S23. Log-dose probit mortality data for three insecticides against transgenic D. melanogaster expressing CYP6CM2, CYP6CM3 or CYP6CM4. [file 12864_2019_6397_MOESM22_ESM.docx]

**Additional file 22: Table S23:** Log-dose probit mortality data for three insecticides against transgenic *D. melanogaster* expressing CYP6CM2, CYP6CM3 or CYP6CM4

| **Chemical** | **Strain** | **LC_50_ Value (95% CI) (mgL^-1^)** | **Slope** | **Tolerance Factor** |
| --- | --- | --- | --- | --- |
| Imidacloprid | Control | 7812 (4400-16068) | 1.393±0.284 | 1 |
|  | CM2 | 951 (462-1939) | 0.974±0.137 | 0.12 |
|  | CM3 | 3218 (1813-6281) | 0.953±0.145 | 0.41 |
|  | CM4 | 1699 (685-4683) | 0.938±0.165 | 0.22 |
| Clothianidin | Control | 123 (62-217) | 2.847±0.333 | 1 |
|  | CM2 | 158 (127-194) | 3.154±0.418 | 1.28 |
|  | CM3 | 97 (59-158) | 1.872±0.211 | 0.78 |
|  | CM4 | 107 (80-142) | 2.746±0.337 | 1.15 |
| Nicotine | Control | 18544 (12992-27349) | 3.114±0.392 | 1 |
|  | CM2 | 22446 (13892-442870 | 2.191±0.316 | 1.21 |
|  | CM3 | 33327 (23370-51925) | 3.893±0.622 | 1.79 |
|  | CM4 | 29037 (21645-41447) | 3.221±0.480 | 1.57 |
