# Supplementary material for: Host plant adaptation in the polyphagous whitefly, Trialeurodes vaporariorum, is associated with transcriptional plasticity and altered sensitivity to insecticides
Source: BMC Genomics. 2019 Dec 19;20:996. doi: 10.1186/s12864-019-6397-3 (PMC6923851; doi:10.1186/s12864-019-6397-3)
Supplement: Supplementary file 23 — Additional file 23: Table S24. Sequence of oligonucleotide primers used in this study. [file 12864_2019_6397_MOESM23_ESM.docx]

**Additional file 23: Table S24**: Sequence of oligonucleotide primers used in this study.

| **Primer Name** | **Sequence (5’-3’)** | **Purpose** |
| --- | --- | --- |
| D099 pUAST F | TCACTGGAACTAGGCTAGCA | Sequence validation of transgenic flies |
| D102 pUAST F | GGATCCAAGCTTGCATGCCTG | Sequence validation of transgenic flies |
| D100 pUAST R | AAAGGCATTCCACCACTGCT | Sequence validation of transgenic flies |
| D101 pUAST R | CCACCACTGCTCCCATTCAT | Sequence validation of transgenic flies |
| NaCh/Para (HK1) F | GACATTGCTCCGAGTCGTT | qPCR housekeeping genes |
| NaCh/Para (HK1) R | AGTGACATAGCGAGAGCGAAT | qPCR housekeeping genes |
| Elongation Factor (HK1) F | GATGGCACGGAGACAATATG | qPCR housekeeping genes |
| Elongation Factor (HK2) R | TTGTCAGTGGGTCTGCTAGG | qPCR housekeeping genes |
| CYP6CM2 F | GGATGGAAAGCAGTTCGGAT | qPCR of CYP6CM2 |
| CYP6CM2 R | CTTTCGGACGTTCATTAAAATTGA | qPCR of CYP6CM2 |
| CYP6CM3 F | AAGTAGTTTCACTGATCTTTATCAT | qPCR of CYP6CM3 |
| CYP6CM3 R | GGAAATCTTGGACGAGATTG | qPCR of CYP6CM3 |
| CYP6CM4 F | GTTGGAAAGTTGTTGAACCAG | qPCR of CYP6CM4 |
| CYP6CM4 R | CACCCTGTTTTAGTGCTTCC | qPCR of CYP6CM4 |
| g58818 F | CGCAAGATACTGTGGATACTTG | qPCR validation of RNA seq. data |
| g58818 R | CCACAGGAGAAATGCTGAAGA | qPCR validation of RNA seq. data |
| g16316 F | CGCAGCCAAACTACCAAATAC | qPCR validation of RNA seq. data |
| g16316 R | GCTCACCTGTGCAACAGTATT | qPCR validation of RNA seq. data |
| g8622 F | GTCGCAGGGATTCTTTTCATTT | qPCR validation of RNA seq. data |
| g8622 R | CGGTGACCTTCAAATCGCAA | qPCR validation of RNA seq. data |
| g20192 f | GGTGAAGGAGTGAATGTGGTT | qPCR validation of RNA seq. data |
| g20192 R | CGCACTCCAGGACACTGTTT | qPCR validation of RNA seq. data |
| g20143 F | CCGAGTCTCCATTGCGTGAA | qPCR validation of RNA seq. data |
| g20143 R | GCCACCAATGTGCTATCCAAA | qPCR validation of RNA seq. data |
| g10064 F | GCCCAAGGAAGTGGACAAAAA | qPCR validation of RNA seq. data |
| g10064 R | CGGTCCATTCCTCGCATTTT | qPCR validation of RNA seq. data |
